# Effects of medical interventions on health-related quality of life in chronic disease – systematic review and meta-analysis of the 19 most common diagnoses

**DOI:** 10.3389/fpubh.2024.1313685

**Published:** 2024-02-06

**Authors:** Franziska Riecke, Leandra Bauer, Hans Polzer, Sebastian Felix Baumbach, Carl Neuerburg, Wolfgang Böcker, Eva Grill, Maximilian Michael Saller

**Affiliations:** ^1^Department of Orthopaedics and Trauma Surgery, Musculoskeletal University Center Munich (MUM), Ludwig-Maximilians-University (LMU) Hospital, Munich, Germany; ^2^Department of Orthopaedics, University Hospital Jena, Campus Eisenberg, Waldkliniken Eisenberg, Eisenberg, Germany; ^3^Institute for Medical Information Processing, Biometry and Epidemiology, LMU, Munich, Germany

**Keywords:** health-related quality of life, chronic disease, EQ-5D, ICD-10, treatment effect size

## Abstract

**Introduction:**

The demographic shift leads to a tremendous increase in age-related diseases, which are often chronic. Therefore, a focus of chronic disease management should be set on the maintenance or even improvement of the patients’ quality of life (QoL). One indicator to objectively measure QoL is the EQ-5D questionnaire, which was validated in a disease- and world region-specific manner. The aim of this study was to conduct a systematic literature review and meta-analysis on the QoL across the most frequent chronic diseases that utilized the EQ-5D and performed a disease-specific meta-analysis for treatment-dependent QoL improvement.

**Materials and methods:**

The most common chronic disease in Germany were identified by their ICD-10 codes, followed by a systematic literature review of these ICD-10 codes and the EQ-5D index values. Finally, out of 10,016 independently -screened studies by two persons, 538 studies were included in the systematic review and 216 studies in the meta-analysis, respectively.

**Results:**

We found significant medium to large effect sizes of treatment effects, i.e., effect size >0.5, in musculoskeletal conditions with the exception of fractures, for chronic depression and for stroke. The effect size did not differ significantly from zero for breast and lung cancer and were significantly negative for fractures.

**Conclusion:**

Our analysis showed a large variation between baseline and post-treatment scores on the EQ-5D health index, depending on the health condition. We found large gains in health-related quality of life mainly for interventions for musculoskeletal disease.

**Systematic review registration:**

https://www.crd.york.ac.uk/prospero/display_record.php?ID=CRD42020150936, PROSPERO identifier CRD42020150936.

## 1 Introduction

The World Health Organization (WHO) assumed that by 2030 more than 1.3 billion and by 2050 more than 2.1 billion people worldwide will be 60 years or older (1). In Germany, 29% of the population were above 60 years in 2021 (2), which is estimated to rise to approximately 33% by 2035 (3). In parallel, the life expectancy has increased by about 2.5 years every decade since World War II (4), and this trend continued until 2019 (5). While the SARS-CoV-2 pandemic led to a slight decrease of global life expectancy at birth, life expectancy is projected to rise to 77.2 years by 2050 (6). For this reason, the WHO initiated the UN Decade of Healthy Aging.^1^

This continuing demographic shift leads to a considerable increase in health conditions that are often chronic or can become chronic if not treated appropriately. Health conditions, such as back pain, are considered chronic if they persist for more than 4 weeks, 3, 6 or 12 months depending on the utilized definition (7). According to a representative survey in Germany (8), 46% of the adult population reported at least one chronic health condition, with cardiovascular (28%) and musculoskeletal conditions (24%) represent the most frequent.

Chronic health conditions are not only frequent but also disabling. Several chronic health conditions that are mostly affecting middle-aged and older adults are a major cause of the growing burden of disease worldwide (9). Ischemic heart disease, stroke, diabetes, COPD and lung cancer, but also musculoskeletal disorders such as injuries and low back pain are among the leading causes contributing to the global burden of disease. To give an example, musculoskeletal disorders were responsible for 150 million disability adjusted life years in 2019. Thus, disability is an increasing concern for public health and national health systems.

Health-related quality of life (HRQoL) is a generally agreed-on concept to determine the impact and burden of disease, injury and disability for the individual and for populations (10). HRQoL is frequently used to measure the treatment effect of therapies because, as a “construct of subjective well-being” (11), it captures the personal experience of the individual in the context of disease. Thus, this is an outcome that is relevant to the individual person. Arguably, therapies for chronic disease should improve survival but they should also improve HRQoL. In the situation of life-threatening disease, however, life-saving therapies might decrease HRQoL, encompassing trade-offs between survival and HRQoL (12). Chronic disease management is likely to have very different effects on HRQoL, depending on the disease state and health condition.

Although HRQoL is a concept that is widely used in very different health situations, there is still no comparison of how treatment affects HRQoL across the most frequent chronic conditions. Objective of our study was to systematically review the literature for treatment effects for the most frequent chronic diseases and quantitatively standardize and summarize effect sizes in a meta-analysis stratified by health condition.

## 2 Materials and methods

### 2.1 Diagnoses

We have defined a chronic disease as a condition that is long-lasting, that cannot be cured completely and that therefore leads to frequent contacts with the health care system (13). To determine the most frequent chronic diseases in Germany, we combined information on documented ICD-10-based diagnosis of the years 2000 to 2018 from public available data resources for ambulatory and in-patient care including rehabilitation. All obtained data was double-checked for plausibility on utilizing GEDA (German Health Update) from the Robert-Koch-Institute. Supplementary Table S1 indicates data sources and their respective scope (14–20).

Diagnoses were excluded if they can be resolved with appropriate treatment, such as cholelithiasis, appendicitis, hernia or pneumonia, or if they are an etiology or a symptom such as atherosclerosis, hypovolemia or lipidaemia, or diagnoses with high prevalence in rehabilitation facilities but with low frequency in ambulatory and acute in-patient care, e.g., cancer of the prostate, multiple sclerosis, or atopic eczema. Obtained During the abstract screening process we additionally identified and included asthma, osteoporosis and fractures as conditions with high impact in disability on a population level.

Asthma (J45) was included, as it is a common medical reason for reference to a rehabilitation institution and the ambulatory segment, and additionally showed a 12-month prevalence as high as diabetes mellitus (around 7%) and even higher lifetime prevalence, when compared to the 12-month prevalence (11.5% vs. 9%) (21).

Osteoporosis with and without fractures (M80/M81) was included, as studies published by the Robert Koch-Institute (RKI) showed a high 12-month prevalence of 7.8% in women and a lifetime prevalence of 8.5% for the whole population (22). In addition, Häussler et al. found that 7.8 million people were affected by osteoporosis in Germany in 2003, which equals a prevalence rate of 26% (23). Also, specific common fracture types were included, as they can lead to chronic impairment.

### 2.2 Outcome: health-related quality of life

For the operationalization of HRQoL, we included all studies that used the five-item questionnaire EQ5D of the EuroQoL Group. This is a generic, cross-disease measure (24, 25) that has been broadly used and validated (26–28). The EQ5D contains 5 dimensions (mobility, self-care, usual activities, pain/discomfort, and anxiety/depression). The EQ-5D questionnaire initially contained 3 levels (EQ5D3L) and was expanded in 2005 to 5 levels (EQ-5D-5L). The levels range from “I have no problems” to “I am unable.” These levels are changed into a 3- or 5-digit number, depending on the number of levels, and represent the health state of the subject. The health states are then converted into the health index, which is a number ranging from ≤0 (variable) to 1 (best possible health state). The health index is valuated depending on nationally representative data. For comparisons we used the health index based on the 3-level or the 5-level version.

### 2.3 Search strategy, screening, and quality assessment

The systematic review was conducted according to the PRISMA guidelines and was registered to PROSPERO (ID: CRD42020150936, 28th of April 2020). We searched *PubMed*, *Web of Science*, *Embase* and *MedLine* (date of search: 16th of October 2021) by combining search terms for the diseases (Supplementary Table S2) “AND” EQ5D. The inclusion criteria were designed in addition to the Population, Intervention, Comparison, Outcome, Study type (PICOS) criteria.

Population: Patients over the age of 18 year; studies engaging in a diagnosis from our derived list of diagnoses, the diagnosis had to be clearly given, either by the ICD-10 code or through thorough and detailed description of the disease that allowed a clear match to an ICD-10 diagnosis.

- Studies included in systematic review: Pre- or post-treatment EQ5D index values
- Studies included in meta-analysis: Pre- and post-treatment EQ5D index values
- Outcome: EQ-5D index used as one of the outcomes
- Study type: Included were all studies presenting primary data independent of the study design

Studies were included in English or German language. Studies were excluded if we had no access to the full text, if the study was abstract only (e.g., a conference abstract), if the study presented no demographic data, if descriptive data were only provided as median, if the diagnosis was not clearly described, if the diagnosis did not fall within our preset definitions, if diagnosis ascertainment was only based on self-report, if the study was using simulated data, if the study was a review, or meta-analysis. We also excluded study protocols without results.

All studies were imported into *Endnote X9*, screened for duplicates electronically and manually. After screening of titles and abstracts, two independent reviewers assessed full texts for eligibility, any discrepancies were resolved by discussion. The main outcome was the EQ-5D index (pre- and/or post-treatment to the last available follow-up). Data was then extracted by two independent reviewers and any discrepancies were resolved by cooperative double-check of the original data of the publication.

Risk of bias analysis was performed for all studies, that were included in the meta-analysis. For randomized controlled studies the RoB2 tool (29) (Supplementary Table S3) and for cohort studies the Newcastle-Ottawa Scale (NOS) (30) (Supplementary Table S4) was used.

### 2.4 Statistical analysis

A study qualified for meta-analysis if the mean ± SD of the EQ-5D index, as well as the participants size could extracted or calculated from each study group (different intervention or observation) of all publication. In case only the (Equation 1) 95% confidence interval (CI) or (Equation 2) standard error (SE) were reported, they were converted to standard deviation (SD) according to the following formula (31):


(1) $$SD=\frac{\mathrm{upper}\;\mathrm{limit}-\mathrm{lower}\;\mathrm{limit}}{3.92}\;x\;\sqrt{N}$$

(2) $$SD=SE\;x\sqrt{N}$$

Collected quantitative data was analyzed with the statistical software R (version 4.2.2), including rstatix (version 0.7.1) for descriptive statistics and metafor (version 3.8–1) for meta-analysis. Data was visualized with ggplot2 (version 3.4.0) and metaviz (version 0.3.1). The effect size (Cohen’s D) within the meta-analysis was calculated utilizing the standardized mean difference (SMD) (32) and a random-effects model. The heterogeneity between the individual studies was determined using a chi-square test. A color family was assigned to every ICD-10 diagnosis group (e.g., musculoskeletal = orange, cardiovascular = green).

## 3 Results

### 3.1 Choice of the most frequent chronic diseases

The chronic diseases were sorted according to their frequencies and the common most frequent diseases were included. From the hospital domain, the three most common ICD-10 codes were angina pectoris (I20), chronic ischaemic heart disease (I25) and atrial fibrillation and flutter (I48). In the rehabilitation facility domain, gonarthrosis (M17), coxarthrosis (M16), and dorsalgia (M54) were the most common. In the outpatient setting, the most cases were reported for dorsalgia (M54), type 2 diabetes mellitus (E11), and chronic ischaemic heart disease (I25).

Altogether, 19 diagnoses were included (Table 1). I10 hypertension was excluded ex post due to the low number of identified studies. I20, I21 and I25 were summarized into one diagnosis group, to represent several ischemic heart diseases. M80/81 were summarized as osteoporosis with and without fractures.

**Table 1:** Included diagnosis with ICD-10 codes and their definitions.

ICD-10	Definition
C34	Malignant neoplasm of bronchus and lung
C50	Malignant neoplasm of breast
E11	Type 2 diabetes mellitus
F33	Recurrent depressive disorder
G40	Epilepsy
I20/I21/I25	Angina pectoris / Acute myocardial infarction / Chronic ischaemic heart disease
I48	Atrial fibrillation and flutter
I50	Heart failure
I63	Cerebral infarction
J44	Other chronic obstructive pulmonary disease
J45	Asthma
M16	Coxarthrosis
M17	Gonarthrosis
M51	Other intervertebral disc disorder
M54	Dorsalgia
M80/M81	Osteoporosis with and without pathological fracture
S52	Fracture of forearm
S72	Fracture of femur
S82	Fracture of lower leg, including ankle

### 3.2 Systematic review

**Figure 1:**
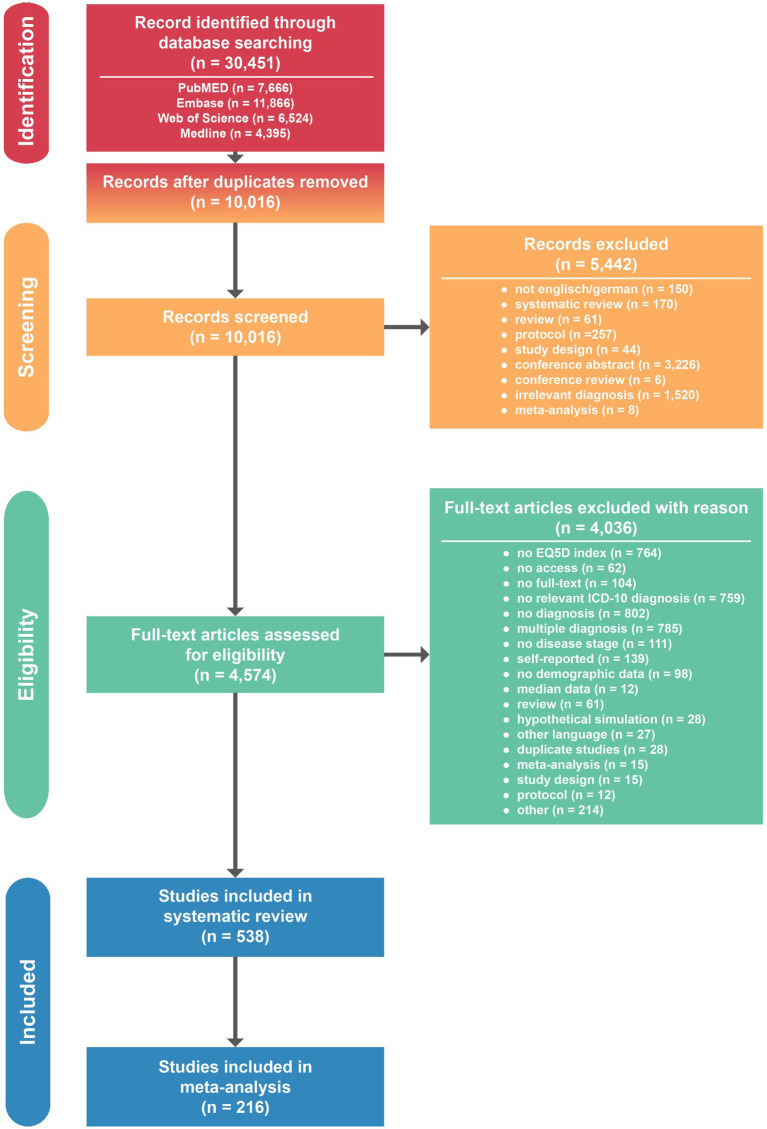
PRISMA flow diagram for study identification and selection.

We identified 30,451 studies of whose 20,435 studies were duplicates. After initial screening, we excluded further 5,442 studies. The remaining 4,574 studies were assessed in full text and 538 studies included in the systematic review. 216 studies were included in meta-analysis (Figure 1).

Quantitative data from all studies was extracted and summarized by ICD-10 code (Table 2). A total of 96 studies were identified for type 2 diabetes mellitus (E11), followed by two musculoskeletal conditions fractures of femur (S72; 60 studies) and gonarthrosis (M17; 39 studies). The smallest number of studies was found for epilepsy (G40; 7 studies) and fractures of forearm (S52; 11 studies). We found an average number of 29 studies per diagnosis.

**Table 2:** Summary of total included studies and studies utilized for meta-analysis.

ICD10	Sum of patients	# of studies	Age of patients [mean ± SD]	# of studies for meta-analysis	% of studies for meta-analysis	Age of patients (studies for meta-analysis) [mean ± SD]
Malignant neoplasm of bronchus and lung (C34)	8,472	17	62.18 ± 7.25	3	17.65	56.60 ± 9.69
Malignant neoplasm of breast (C50)	18,769	38	57.46 ± 8.29	10	26.32	58.38 ± 8.44
Type 2 diabetes mellitus (E11)	121,711	96	61.35 ± 5.31	14	14.58	61.37 ± 4.10
Recurrent depressive disorder (F33)	8,891	28	46.31 ± 10.58	17	60.71	45.28 ± 8.37
Epilepsy (G40)	2,550	7	37.23 ± 3.31	2	28.57	37.45 ± 1.55
Angina pectoris / Acute myocardial infarction / Chronic ischaemic heart disease (I20/I21/I25)	61,339	26	63.53 ± 4.57	9	34.62	62.03 ± 2.82
Atrial fibrillation and flutter (I48)	13,065	17	65.19 ± 6.38	6	35.29	64.52 ± 6.18
Heart failure (I50)	21,286	27	71.58 ± 7.44	12	44.44	74.42 ± 7.31
Cerebral infarction (I63)	9,188	13	65.39 ± 4.29	6	46.15	64.67 ± 5.70
Other chronic obstructive pulmonary disease (J44)	2,261,814	15	67.00 ± 3.91	2	13.33	68.20 ± 2.26
Asthma (J45)	18,075	18	46.02 ± 6.46	3	16.67	51.59 ± 5.11
Coxarthrosis (M16)	245,850	34	65.78 ± 3.80	23	67.65	65.27 ± 3.74
Gonarthrosis (M17)	105,996	39	66.94 ± 4.14	26	66.67	66.41 ± 4.70
Other intervertebral disc disorder (M51)	116,515	36	45.77 ± 6.08	20	55.56	46.29 ± 6.48
Dorsalgia (M54)	2,651	17	46.71 ± 11.15	9	52.94	47.75 ± 10.00
Osteoporosis with and without pathological fracture (M80/M81)	21,594	24	69.91 ± 4.21	12	50.00	72.16 ± 4.11
Fracture of forearm (S52)	2,612	11	62.51 ± 6.10	5	45.45	63.71 ± 7.42
Fracture of femur (S72)	39,737	60	77.21 ± 10.95	37	61.67	75.02 ± 12.85
Fracture of lower leg, including ankle (S82)	2,373	20	45.89 ± 8.96	3	15.00	46.54 ± 10.00
Total	3,082,48	543^1^		219^2^		

1 5 studies contained data on 2 diagnoses and therefore count double.

2 3 studies contained data on 2 diagnoses and therefore count double.

A total of 224 studies were observational cohort studies, followed by randomized controlled trials (140 studies), cross-sectional studies (105 studies) and 4 case control studies. A total of 66 studies did not report a study type. Overall, we found the most participants with 2,261,814 for other chronic obstructive pulmonary disease (J44), followed by coxarthrosis (M16, 245,850 participants) and type 2 diabetes mellitus (E11, 121,711 participants). The fewest participants were found for fracture of lower leg, including ankle (S82, 2,373 participants) and epilepsy (G40, 2,550 participants). Patients with epilepsy (G40) had the lowest mean age (37.2 ± 3.3 years), patients with fracture of femur (S72) had the highest mean age (77.2 ± 11.0 years) (Table 2).

### 3.3 Analysis of pre- and post-treatment EQ-5D indices of the most frequent chronic diseases

**Figure 2:**
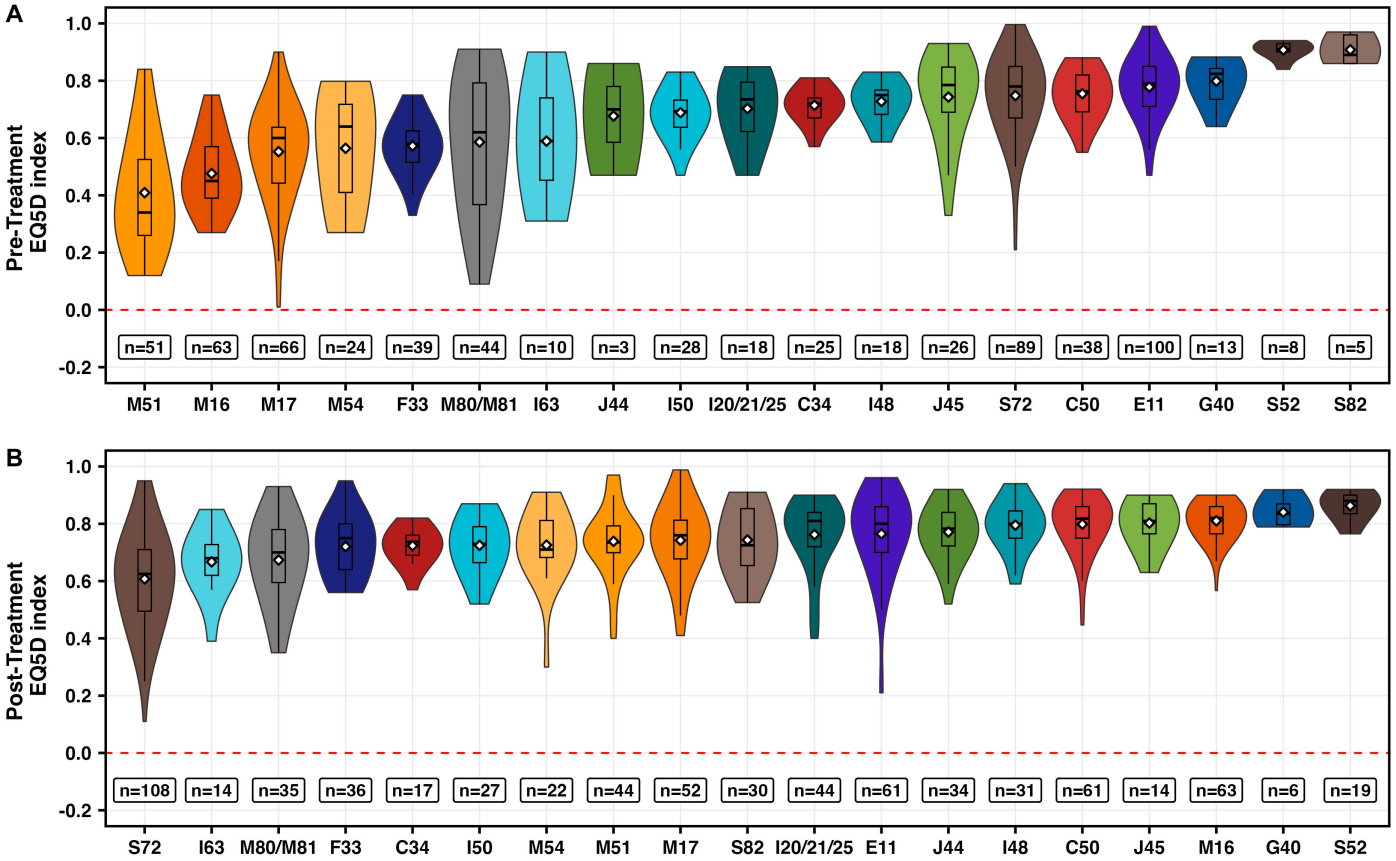
Visualization of pre- **(A)** and post-treatment **(B)** EQ-5D index values per ICD-10 code and participant group. Box plots represent the median, quartiles, range and mean (white diamond). n equals the number of participant groups.

In total, we included 400 studies with 668 participant groups that reported pre-treatment EQ-5D index values and 718 participant groups derived from 409 studies that reported post-treatment EQ-5D index values. Interestingly, especially patients with musculoskeletal conditions such as other intervertebral disorder (M51), coxarthrosis (M16), gonarthrosis (M17), or dorsalgia (M54) reported the lowest average EQ-5D index (Figure 2A and Supplementary Table S5). In contrast, type 2 diabetes mellitus (E11) or epilepsy (G40) showed a very good mean pretreatment EQ-5D index. Moreover, patients whose QoL was not affected before a fracture of the forearm or lower leg including ankle (S52/S82) also stated a relatively good state of health (Figure 2A and Supplementary Table S5).

After a medical intervention, patients with a fracture of the femur (S72), cerebral infarction (I63), or osteoporosis with and without pathological fracture (M80/M81) reported the worst QoL (Figure 2B and Supplementary Table S3). In contrast, patients with a fracture of the forearm (S52), epilepsy (G40), or coxarthrosis (M16) reported the best QoL (Figure 2B and Supplementary Table S5).

The largest range was identified for gonarthrosis (M17) with pre-treatment EQ-5D indices ranging from 0.01 (33) to 0.9 (34) (Figure 2A). The largest range of post-treatment EQ-5D indices was found in fractures of the femur (S72), with EQ-5D index ranging from 0.11 to 0.95 (35) (Figure 2B).

### 3.4 EQ-5D specific meta-analysis for the most frequent chronic diseases

**Figure 3:**
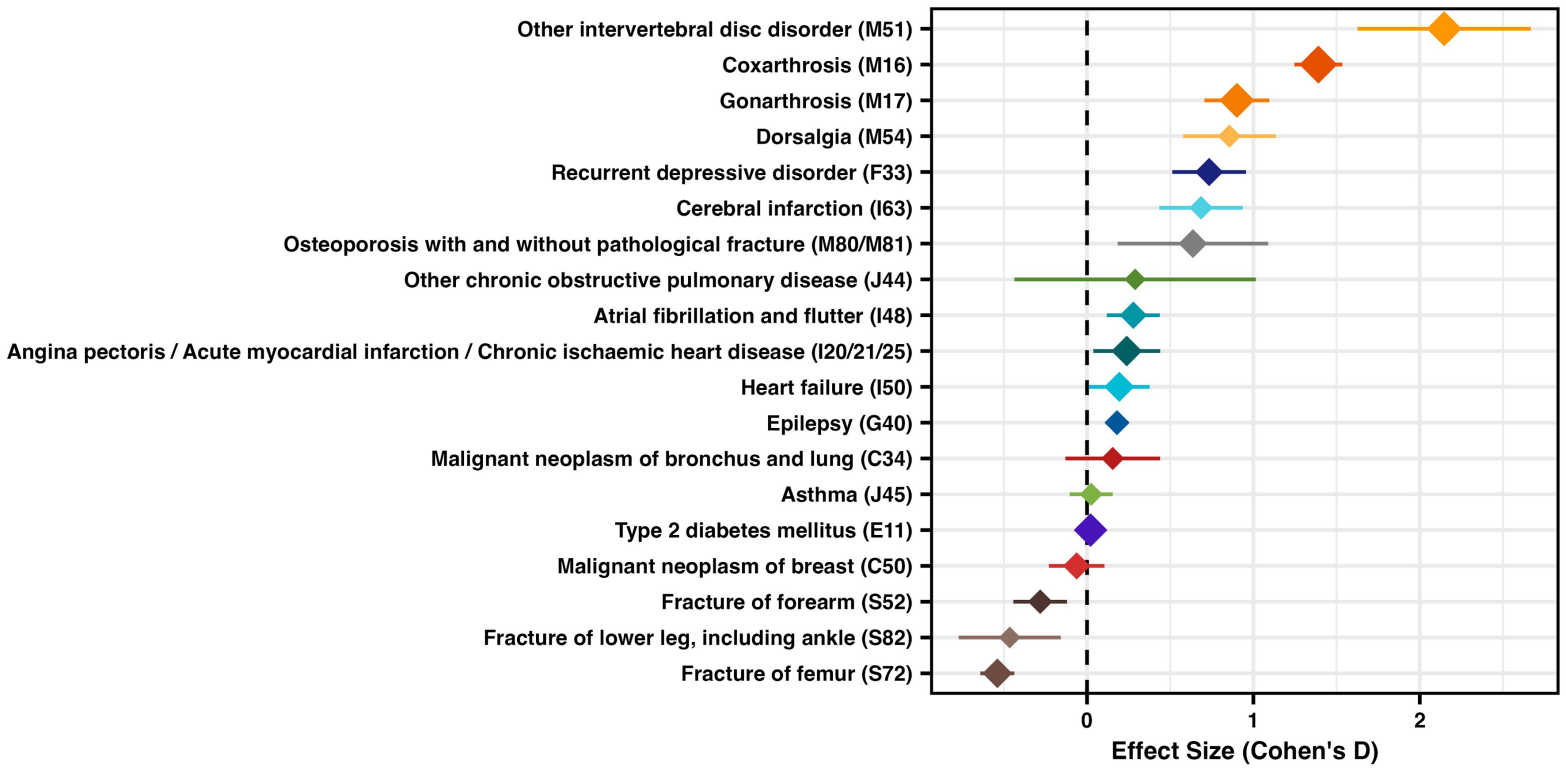
Effect size (Cohen’s D) for studies with pre and post values of EQ-5D, with mean effect size and 95% confidence interval, diamond size represents study size as log2 (total sum of all patients)*0.5.

We identified 216 studies with 377 participant groups. The largest positive effect size was identified for musculoskeletal orthopedic conditions such as intervertebral disk disorders (M51, Cohen’s D) followed by coxarthrosis (M16) (Figure 3 and Supplementary Table S6). Musculoskeletal trauma such as fractures of the femur (S72), fractures of the lower leg (S82) or fractures of the forearm (S52) as well as breast cancer (C50) had an overall negative effect size (Figure 3 and Supplementary Table S6). All other diagnoses showed a positive effect size (Figure 3 and Supplementary Table S6).

**Figure 4:**
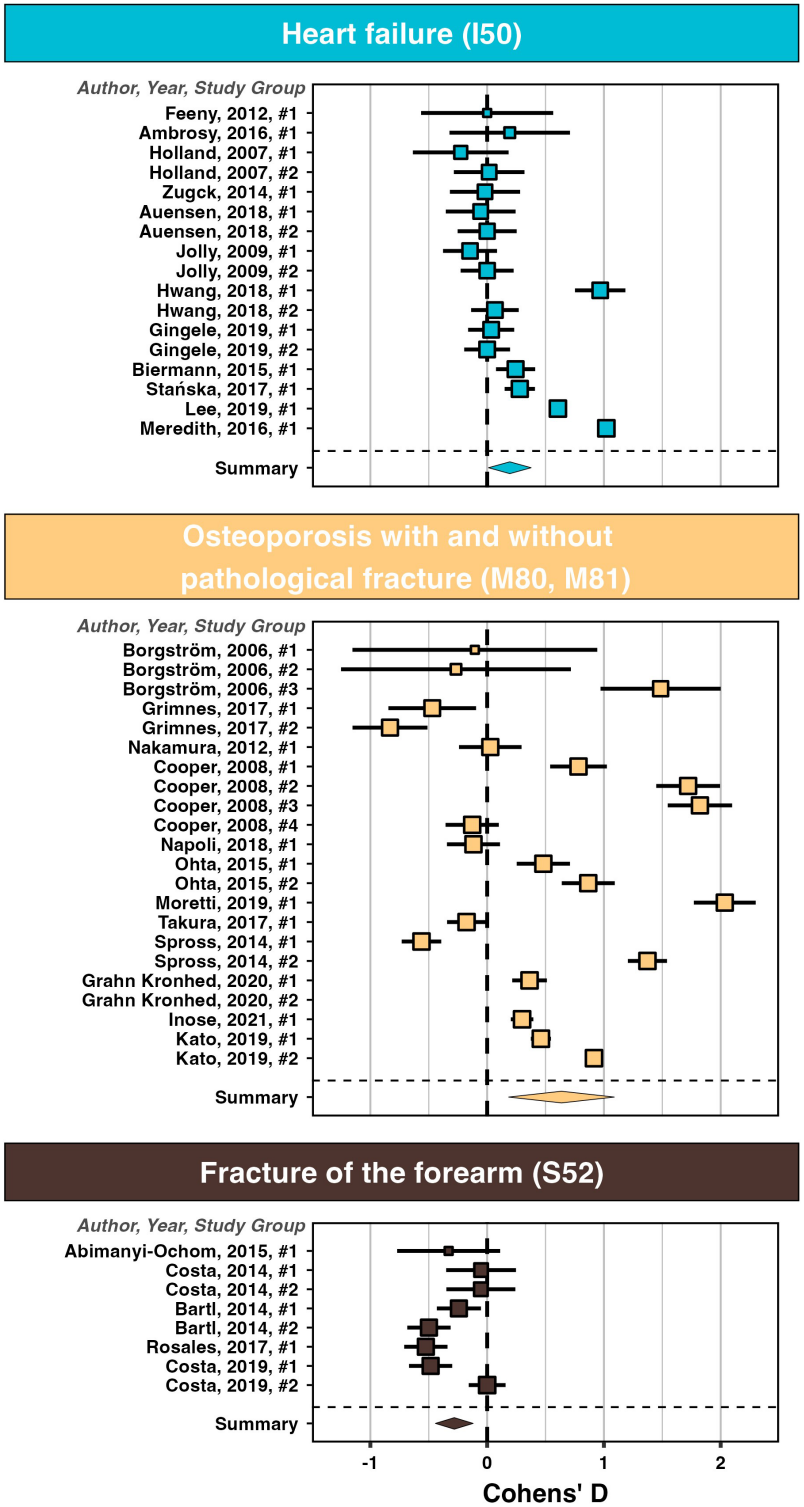
Forest-plots with the effect size (Cohen’s D) for studies with a pre and a post EQ-5D for heart failure (I50), osteoporosis with and without pathological fracture (M80, M81), as well as fracture of forearm (S52).

To visualize effect size within diagnoses we show three exemplary forest plots with the individual studies in Figure 4. All other forest plots are available in the Supplementary Files.

## 4 Discussion

To our knowledge this is the first comprehensive systematic review and meta-analysis that compares treatment effects on health-related quality of life (HRQoL) across the most frequently encountered chronic diagnoses in ambulatory, hospital and rehabilitation situations. The EQ-5D is a widely accepted measure to assess HRQoL, and consequently, we retrieved many studies that had used the EQ-5D to quantify patient-reported outcomes. Initial and post-treatment values of the EQ-5D health index varied broadly within and among diagnoses, arguably also depending on inclusion criteria of the single studies, but we could identify several patterns that illustrate the differing impact of therapy on HRQoL in chronic disease. We found significant medium to large effect sizes of treatment effects, i.e., effect size >0.5 (36), in musculoskeletal conditions with the exception of fractures, for chronic depression and for stroke. The effect size did not differ significantly from zero for breast and lung cancer and were significantly negative for fractures.

Patients with musculoskeletal conditions reported the widest health index range in our study with low mean pre-treatment values. The wide range of pre-treatment health indices for gonarthrosis could be a result of different disease stages. Individuals treated with a non-surgical approach, and therefore presumably at an earlier stage of the disease, may have a higher pre-treatment HRQoL, whereas patients with long-standing disease are most likely to have a lower pre-treatment health index requiring surgery. This is in line with findings from other multinational comparisons (37). To give an example, for osteoarthritis, our study found pre-treatment health index values quite similar to other comparable studies (38). Likewise, a recent meta-analysis showed low preoperative EQ-5D health index values for lumbar spondylosis and knee and hip osteoarthritis (39). Effect sizes for treatment effects in knee and hip replacement were large in our study, which is plausible and consistent with the objective performance criteria expected for total joint replacement (40). Also, a cross-diagnosis meta-analysis for surgical interventions found that lumbar spine surgery and joint arthroplasty yielded the largest gains in HRQoL (39). Recent studies confirmed this high responsiveness of the EQ-5D in knee arthroplasty (41). It is still unclear whether patient characteristics have an influence on changes in the EQ-5D Index after arthroplasty, a clear effect has so far only been shown for body mass index (42). The negative effect size shown in our study for femur fracture confirms other meta-analytic findings that older patients with hip fracture in particular do not experience a full recovery of their HRQoL to baseline values (43). Individuals with fractures of lower leg and forearm are likely to be younger and consequently at a high pre-treatment health index value. For example, a meta-analysis of upper limb surgery found little evidence of health index gains (44).

The large effect sizes for depression and anxiety in our study are consistent with recent findings confirming that the EQ-5D is sensitive to changes in self-reported symptoms (45).

The moderate effect sizes found in our study for stroke are paralleled by findings from a Cochrane umbrella meta-analysis of intervention effects on upper limb mobility in stroke patients (46). While the authors of the Cochrane review concluded that there was little robust evidence for the effectiveness of established interventions for upper limb function in stroke, the EQ-5D generally showed good responsiveness in stroke trials (47).

Interestingly, our study found that patients with breast cancer had high pre-treatment values of the health index. This aligns with health indices ranging from 0.77 to 0.92 according to breast cancer stage reported for Chinese women (48). Likewise, the health index range found in our study for lung cancer is similar to the mean stage-dependent values ranging from 0.69 to 0.78 reported from a recent meta-analysis (49), with patients with progressed disease reporting lower values. HRQoL effect sizes were generally small in advanced cancer trials (50).

To put our findings into context, it might be helpful to look closer at the dimensions of the health index. The EQ-5D evaluates mobility, self-care, activities of daily living, pain, and anxiety/depression. Although responsiveness and relevance of the health index has been shown for many chronic diseases, effect size will arguably be larger if the health condition affects several domains at once, as seen in musculoskeletal disease, such as joint osteoarthritis. Also, effect sizes will be larger if the baseline pre-treatment value is low. At the same time, newly diagnosed low-stage neoplastic disease may affect only a few domains of the health index, where the EQ-5D is less responsive to typical problems such as cancer fatigue. Researchers might want to additionally rely on condition specific HRQoL measures that are designed to capture the full experience of disease and are less prone to ceiling effects. Nevertheless, the generic EQ-5D has a valid and important role. Patient-reported generic outcomes such as the health index are highly relevant because they indicate individual preference of patient-relevant outcomes. Also, the cross-condition approach is essential to capture and appraise burden of disease. Thus, the health index allows to plan treatment strategies and to adapt them if necessary. In progressive disease situations, this also widens the decision-making space beyond survival. Still, it has to be kept in mind that individual preference as monitored by the EQ-5D health index may vary by health condition, by individual characteristics such as age and sex, or by country-specific value sets (51).

Our study is a very broad meta-analysis, and some limitations must be discussed. We included the most frequent diagnoses presented in the German health system which is based on a statutory health insurance funds that currently cover almost 90% of the population with free access to services that largely exceed the essential. This certainly affects the frequency and type of consultations and intervention choices. Our selection of chronic diagnoses, however, are in agreement with the most frequent chronic conditions, namely cardiovascular diseases, stroke, diabetes, cancer, chronic obstructive pulmonary disease, musculoskeletal conditions, mental health conditions (3).

Our analysis showed a large variation between baseline and post-treatment scores on the EQ-5D health index, depending on the health condition. While treatment gains in health-related quality of life were quite large for degenerative musculoskeletal diseases, effect sizes were small in musculoskeletal injuries Type 2 diabetes mellitus, cardiovascular diseases or COPD. This may be an inherent effect of the generic EQ-5D health index that puts specific emphasis on mobility, self-care and pain, which are typical problems encountered in musculoskeletal disease but to a lesser extent, e.g., in metabolic disease. To investigate treatment effects, it is necessary to fully capture the patients’ experience. Still, patient-reported generic outcome measures such as the health index are highly relevant because they indicate individual preference of patient-relevant outcomes, capture burden of disease across health conditions, allow to make treatment decisions and to adapt treatment strategies if necessary.

## Data availability statement

The original contributions presented in the study are included in the article/Supplementary material, further inquiries can be directed to the corresponding author.

## Author contributions

FR: Conceptualization, Data curation, Formal analysis, Investigation, Methodology, Validation, Writing – original draft. LB: Data curation, Formal analysis, Methodology, Software, Validation, Writing – original draft. HP: Conceptualization, Writing – review & editing. SB: Writing – review & editing. CN: Writing – review & editing. WB: Writing – review & editing. EG: Formal analysis, Methodology, Supervision, Validation, Writing – original draft. MS: Conceptualization, Data curation, Formal analysis, Investigation, Methodology, Project administration, Software, Supervision, Validation, Writing – original draft.

## Footnotes

^1^ https://www.who.int/initiatives/decade-of-healthy-ageing
