# Supplementary material for: Effects of medical interventions on health-related quality of life in chronic disease – systematic review and meta-analysis of the 19 most common diagnoses
Source: Front Public Health. 2024 Feb 6;12:1313685. doi: 10.3389/fpubh.2024.1313685 (PMC10878130; doi:10.3389/fpubh.2024.1313685)
Supplement: Supplementary file 1 [file Table_1.docx]

Tab.S1

| Institution | Type | Scope | Source |
| --- | --- | --- | --- |
| Allgemeine Ortskrankenkasse (AOK) | Statutory health insurance | Ambulatory and hospital | 14 |
| Barmer Ersatzkasse (Barmer) | Statutory health insurance | Ambulatory and hospital | 15 |
| Betriebskrankenkasse (BKK) | Statutory health insurance | Ambulatory and hospital | 16 |
| Statistisches Bundesamt | Federal Statistical Office of Germany | Ambulatory, hospital and rehabilitation | 17 |
| Destatis | Federal Statistical Office of Germany | Ambulatory, hospital and rehabilitation | 18 |
| National Association of Statutory Health Insurance Physicians | Political lobby for ambulatory physicians and psychotherapists | Ambulatory | 19 |
| Central Research Institute of Ambulatory Health Care in Germany | Research institute | Ambulatory, hospital and rehabilitation | 20 |
| Robert Koch Institute (RKI) | Federal institute for disease surveillance and prevention | Ambulatory, hospital and rehabilitation | https://www.rki.de/ |
