# Supplementary material for: Effects of medical interventions on health-related quality of life in chronic disease – systematic review and meta-analysis of the 19 most common diagnoses
Source: Front Public Health. 2024 Feb 6;12:1313685. doi: 10.3389/fpubh.2024.1313685 (PMC10878130; doi:10.3389/fpubh.2024.1313685)
Supplement: Supplementary file 3 [file Table_3.docx]

Tab.S3

| ICD-10 | time point of EQ-5D | n | min | max | median | iqr | mean | sd | se | ci |
| --- | --- | --- | --- | --- | --- | --- | --- | --- | --- | --- |
| C34 | pre | 25 | 0.57 | 0.81 | 0.72 | 0.07 | 0.71 | 0.06 | 0.01 | 0.03 |
| C34 | post | 17 | 0.57 | 0.82 | 0.74 | 0.07 | 0.72 | 0.06 | 0.02 | 0.03 |
| C50 | pre | 38 | 0.55 | 0.88 | 0.76 | 0.13 | 0.76 | 0.08 | 0.01 | 0.03 |
| C50 | post | 61 | 0.45 | 0.92 | 0.82 | 0.11 | 0.80 | 0.10 | 0.01 | 0.02 |
| E11 | pre | 100 | 0.47 | 0.99 | 0.79 | 0.14 | 0.78 | 0.10 | 0.01 | 0.02 |
| E11 | post | 61 | 0.21 | 0.96 | 0.80 | 0.16 | 0.77 | 0.15 | 0.02 | 0.04 |
| F33 | pre | 39 | 0.33 | 0.75 | 0.58 | 0.11 | 0.57 | 0.10 | 0.02 | 0.03 |
| F33 | post | 36 | 0.56 | 0.95 | 0.75 | 0.16 | 0.72 | 0.10 | 0.02 | 0.03 |
| G40 | pre | 13 | 0.64 | 0.88 | 0.82 | 0.11 | 0.80 | 0.07 | 0.02 | 0.04 |
| G40 | post | 6 | 0.79 | 0.92 | 0.84 | 0.07 | 0.84 | 0.05 | 0.02 | 0.05 |
| I20/21/25 | pre | 18 | 0.47 | 0.85 | 0.74 | 0.17 | 0.70 | 0.11 | 0.03 | 0.06 |
| I20/21/25 | post | 44 | 0.40 | 0.90 | 0.81 | 0.12 | 0.76 | 0.13 | 0.02 | 0.04 |
| I48 | pre | 18 | 0.59 | 0.83 | 0.75 | 0.09 | 0.73 | 0.07 | 0.02 | 0.04 |
| I48 | post | 31 | 0.59 | 0.94 | 0.80 | 0.10 | 0.80 | 0.09 | 0.02 | 0.03 |
| I50 | pre | 28 | 0.47 | 0.83 | 0.69 | 0.10 | 0.69 | 0.09 | 0.02 | 0.03 |
| I50 | post | 27 | 0.52 | 0.87 | 0.73 | 0.13 | 0.73 | 0.10 | 0.02 | 0.04 |
| I63 | pre | 10 | 0.31 | 0.90 | 0.59 | 0.29 | 0.59 | 0.20 | 0.06 | 0.15 |
| I63 | post | 14 | 0.39 | 0.85 | 0.68 | 0.11 | 0.67 | 0.12 | 0.03 | 0.07 |
| J44 | pre | 3 | 0.47 | 0.86 | 0.70 | 0.20 | 0.68 | 0.20 | 0.11 | 0.49 |
| J44 | post | 34 | 0.52 | 0.92 | 0.78 | 0.12 | 0.77 | 0.09 | 0.02 | 0.03 |
| J45 | pre | 26 | 0.33 | 0.93 | 0.79 | 0.16 | 0.74 | 0.15 | 0.03 | 0.06 |
| J45 | post | 14 | 0.63 | 0.90 | 0.81 | 0.11 | 0.80 | 0.08 | 0.02 | 0.05 |
| M16 | pre | 63 | 0.27 | 0.75 | 0.45 | 0.18 | 0.48 | 0.12 | 0.02 | 0.03 |
| M16 | post | 63 | 0.57 | 0.90 | 0.82 | 0.10 | 0.81 | 0.07 | 0.01 | 0.02 |
| M17 | pre | 66 | 0.01 | 0.90 | 0.60 | 0.20 | 0.55 | 0.16 | 0.02 | 0.04 |
| M17 | post | 52 | 0.41 | 0.99 | 0.76 | 0.14 | 0.74 | 0.13 | 0.02 | 0.04 |
| M51 | pre | 51 | 0.12 | 0.84 | 0.34 | 0.27 | 0.41 | 0.19 | 0.03 | 0.05 |
| M51 | post | 46 | 0.40 | 1.00 | 0.74 | 0.10 | 0.75 | 0.13 | 0.02 | 0.04 |
| M54 | pre | 24 | 0.27 | 0.80 | 0.64 | 0.31 | 0.56 | 0.19 | 0.04 | 0.08 |
| M54 | post | 22 | 0.30 | 0.91 | 0.71 | 0.13 | 0.73 | 0.13 | 0.03 | 0.06 |
| M80/M81 | pre | 44 | 0.09 | 0.91 | 0.62 | 0.43 | 0.59 | 0.23 | 0.04 | 0.07 |
| M80/M81 | post | 35 | 0.35 | 0.93 | 0.70 | 0.19 | 0.67 | 0.14 | 0.02 | 0.05 |
| S52 | pre | 8 | 0.84 | 0.94 | 0.91 | 0.03 | 0.91 | 0.03 | 0.01 | 0.03 |
| S52 | post | 19 | 0.77 | 0.92 | 0.88 | 0.07 | 0.86 | 0.05 | 0.01 | 0.02 |
| S72 | pre | 89 | 0.21 | 1.00 | 0.78 | 0.18 | 0.75 | 0.14 | 0.02 | 0.03 |
| S72 | post | 108 | 0.11 | 0.95 | 0.63 | 0.22 | 0.61 | 0.16 | 0.02 | 0.03 |
| S82 | pre | 7 | 0.86 | 1.00 | 0.96 | 0.11 | 0.93 | 0.06 | 0.02 | 0.06 |
| S82 | post | 32 | 0.53 | 1.00 | 0.74 | 0.21 | 0.76 | 0.13 | 0.02 | 0.05 |
