# Supplementary material for: Effects of medical interventions on health-related quality of life in chronic disease – systematic review and meta-analysis of the 19 most common diagnoses
Source: Front Public Health. 2024 Feb 6;12:1313685. doi: 10.3389/fpubh.2024.1313685 (PMC10878130; doi:10.3389/fpubh.2024.1313685)
Supplement: Supplementary file 4 [file Table_4.docx]

Tab.S4

|  |  |  |  |
| --- | --- | --- | --- |
| M51 | 2.15 | 1.63 | 2.67 |
| M16 | 1.39 | 1.25 | 1.53 |
| M17 | 0.9 | 0.71 | 1.1 |
| M54 | 0.86 | 0.58 | 1.13 |
| F33 | 0.73 | 0.51 | 0.96 |
| I63 | 0.69 | 0.43 | 0.94 |
| M80/M81 | 0.64 | 0.18 | 1.09 |
| J44 | 0.29 | -0.44 | 1.02 |
| I48 | 0.28 | 0.12 | 0.44 |
| I20/21/25 | 0.24 | 0.04 | 0.44 |
| I50 | 0.19 | 0.01 | 0.38 |
| G40 | 0.18 | 0.11 | 0.25 |
| C34 | 0.15 | -0.13 | 0.44 |
| J45 | 0.03 | -0.1 | 0.15 |
| E11 | 0.02 | -0.05 | 0.09 |
| C50 | -0.06 | -0.23 | 0.11 |
| S52 | -0.28 | -0.44 | -0.12 |
| S82 | -0.46 | -0.77 | -0.16 |
| S72 | -0.54 | -0.64 | -0.44 |
