# Supplementary material for: Effects of medical interventions on health-related quality of life in chronic disease – systematic review and meta-analysis of the 19 most common diagnoses
Source: Front Public Health. 2024 Feb 6;12:1313685. doi: 10.3389/fpubh.2024.1313685 (PMC10878130; doi:10.3389/fpubh.2024.1313685)
Supplement: Supplementary file 10 [file Data_Sheet_1.ZIP › Frontiers_Supplementary_Figures/Riecke et al._Fig.S1A_C34.pdf]

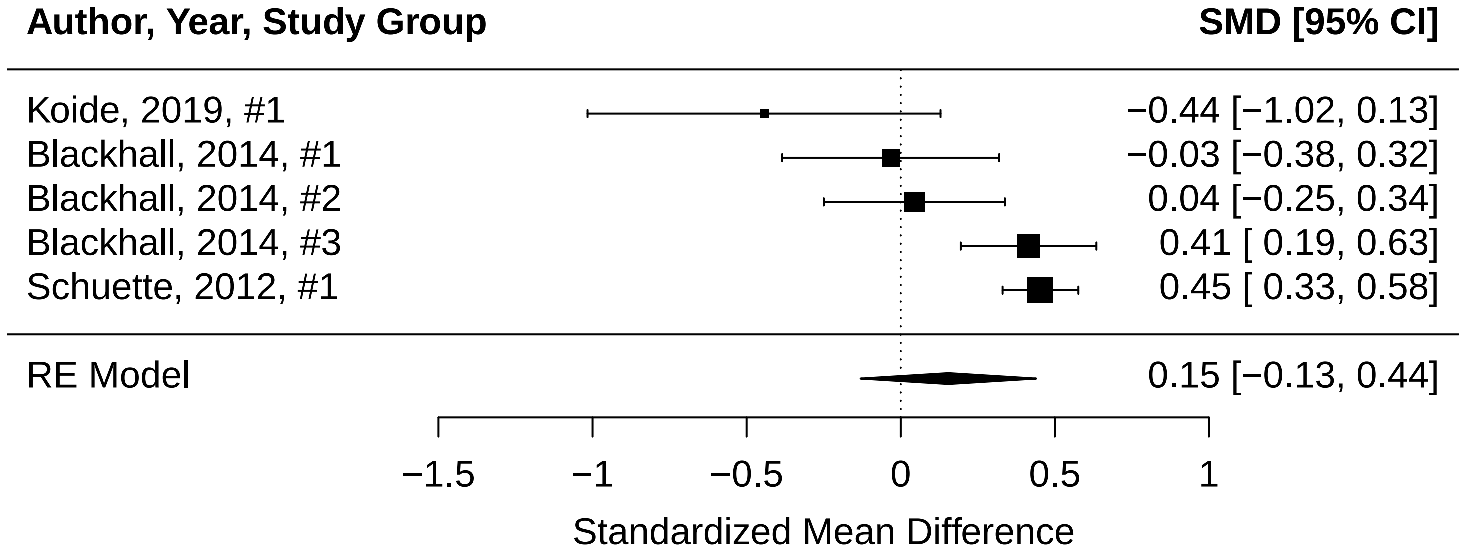

Author, Year, Study Group
SMD [95% CI]
Koide, 2019, #1
−0.44 [−1.02, 0.13]
Blackhall, 2014, #1
−0.03 [−0.38, 0.32]
Blackhall, 2014, #2
0.04 [−0.25, 0.34]
Blackhall, 2014, #3
0.41 [0.19, 0.63]
Schuette, 2012, #1
0.45 [0.33, 0.58]
RE Model
0.15 [−0.13, 0.44]
−1.5
−1
−0.5
0
0.5
1
Standardized Mean Difference
