# Supplementary material for: Effects of medical interventions on health-related quality of life in chronic disease – systematic review and meta-analysis of the 19 most common diagnoses
Source: Front Public Health. 2024 Feb 6;12:1313685. doi: 10.3389/fpubh.2024.1313685 (PMC10878130; doi:10.3389/fpubh.2024.1313685)
Supplement: Supplementary file 10 [file Data_Sheet_1.ZIP › Frontiers_Supplementary_Figures/Riecke et al._Fig.S1E_G40.pdf]

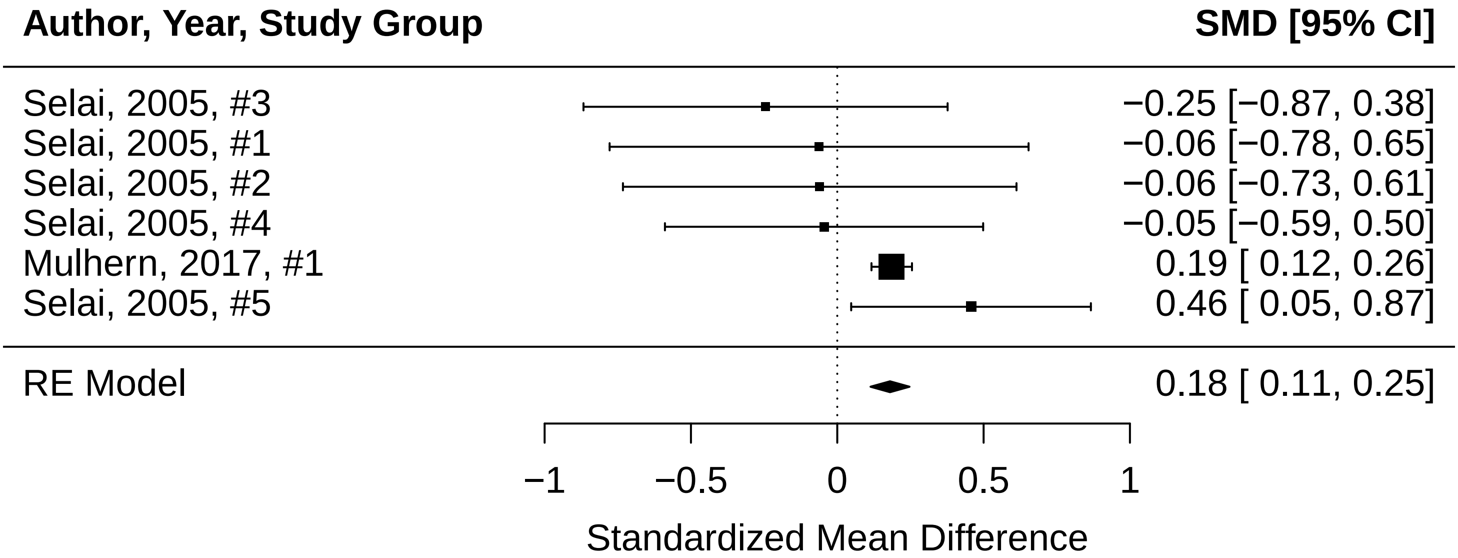
Author, Year, Study Group
SMD [95% CI]
Selai, 2005, #3
−0.25 [−0.87, 0.38]
Selai, 2005, #1
−0.06 [−0.78, 0.65]
Selai, 2005, #2
−0.06 [−0.73, 0.61]
Selai, 2005, #4
−0.05 [−0.59, 0.50]
Mulhern, 2017, #1
0.19 [0.12, 0.26]
Selai, 2005, #5
0.46 [0.05, 0.87]
RE Model
0.18 [0.11, 0.25]
−1
−0.5
0
0.5
1
Standardized Mean Difference
