# Supplementary material for: Effects of medical interventions on health-related quality of life in chronic disease – systematic review and meta-analysis of the 19 most common diagnoses
Source: Front Public Health. 2024 Feb 6;12:1313685. doi: 10.3389/fpubh.2024.1313685 (PMC10878130; doi:10.3389/fpubh.2024.1313685)
Supplement: Supplementary file 10 [file Data_Sheet_1.ZIP › Frontiers_Supplementary_Figures/Riecke et al._Fig.S1G_I48.pdf]

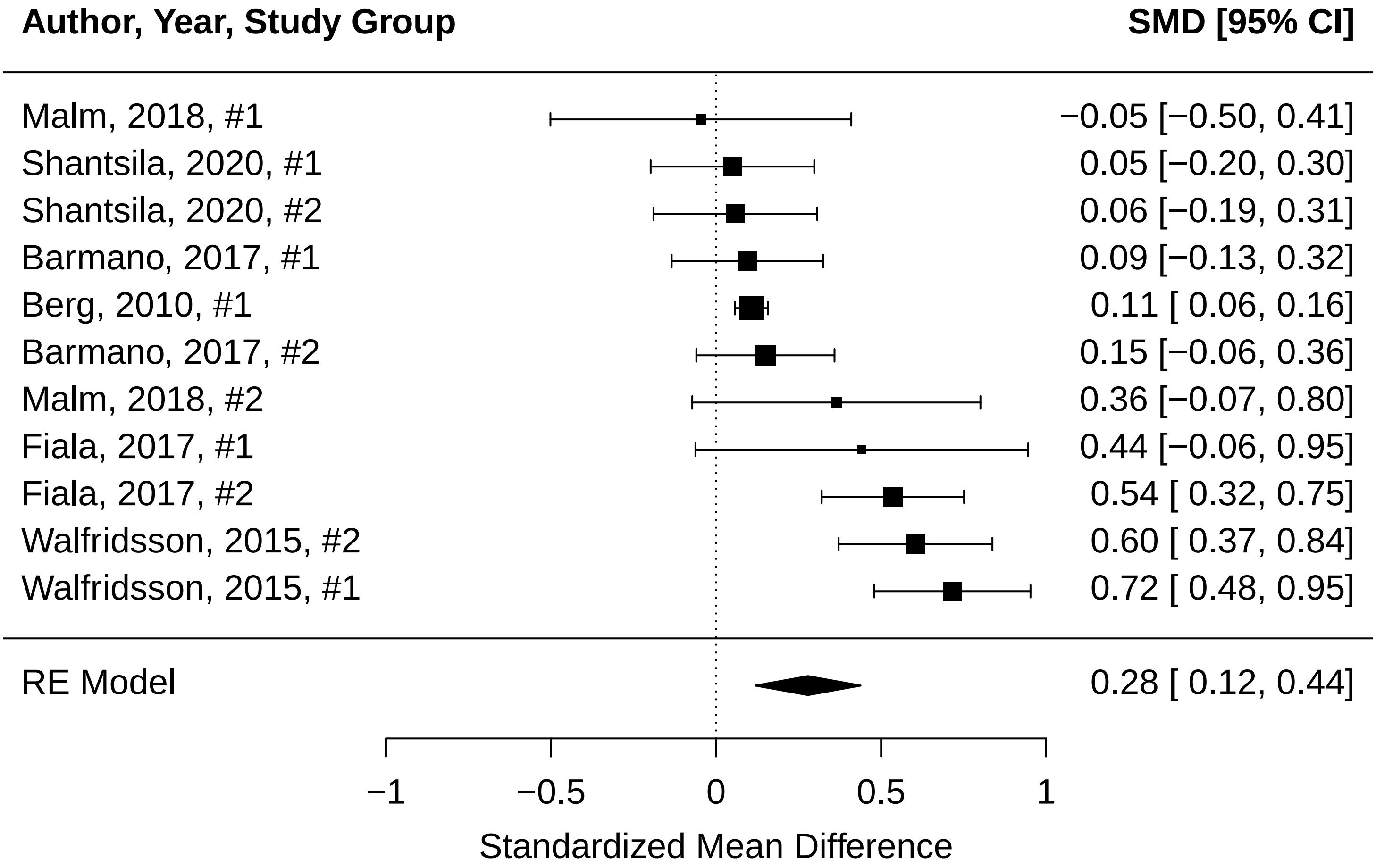
Author, Year, Study Group
SMD [95% CI]
Malm, 2018, #1
−0.05 [−0.50, 0.41]
Shantsila, 2020, #1
0.05 [−0.20, 0.30]
Shantsila, 2020, #2
0.06 [−0.19, 0.31]
Barmano, 2017, #1
0.09 [−0.13, 0.32]
Berg, 2010, #1
0.11 [0.06, 0.16]
Barmano, 2017, #2
0.15 [−0.06, 0.36]
Malm, 2018, #2
0.36 [−0.07, 0.80]
Fiala, 2017, #1
0.44 [−0.06, 0.95]
Fiala, 2017, #2
0.54 [0.32, 0.75]
Walfridsson, 2015, #2
0.60 [0.37, 0.84]
Walfridsson, 2015, #1
0.72 [0.48, 0.95]
RE Model
0.28 [0.12, 0.44]
−1
−0.5
0
0.5
1
Standardized Mean Difference
