# Supplementary material for: Effects of medical interventions on health-related quality of life in chronic disease – systematic review and meta-analysis of the 19 most common diagnoses
Source: Front Public Health. 2024 Feb 6;12:1313685. doi: 10.3389/fpubh.2024.1313685 (PMC10878130; doi:10.3389/fpubh.2024.1313685)
Supplement: Supplementary file 10 [file Data_Sheet_1.ZIP › Frontiers_Supplementary_Figures/Riecke et al._Fig.S1R_S72.pdf]

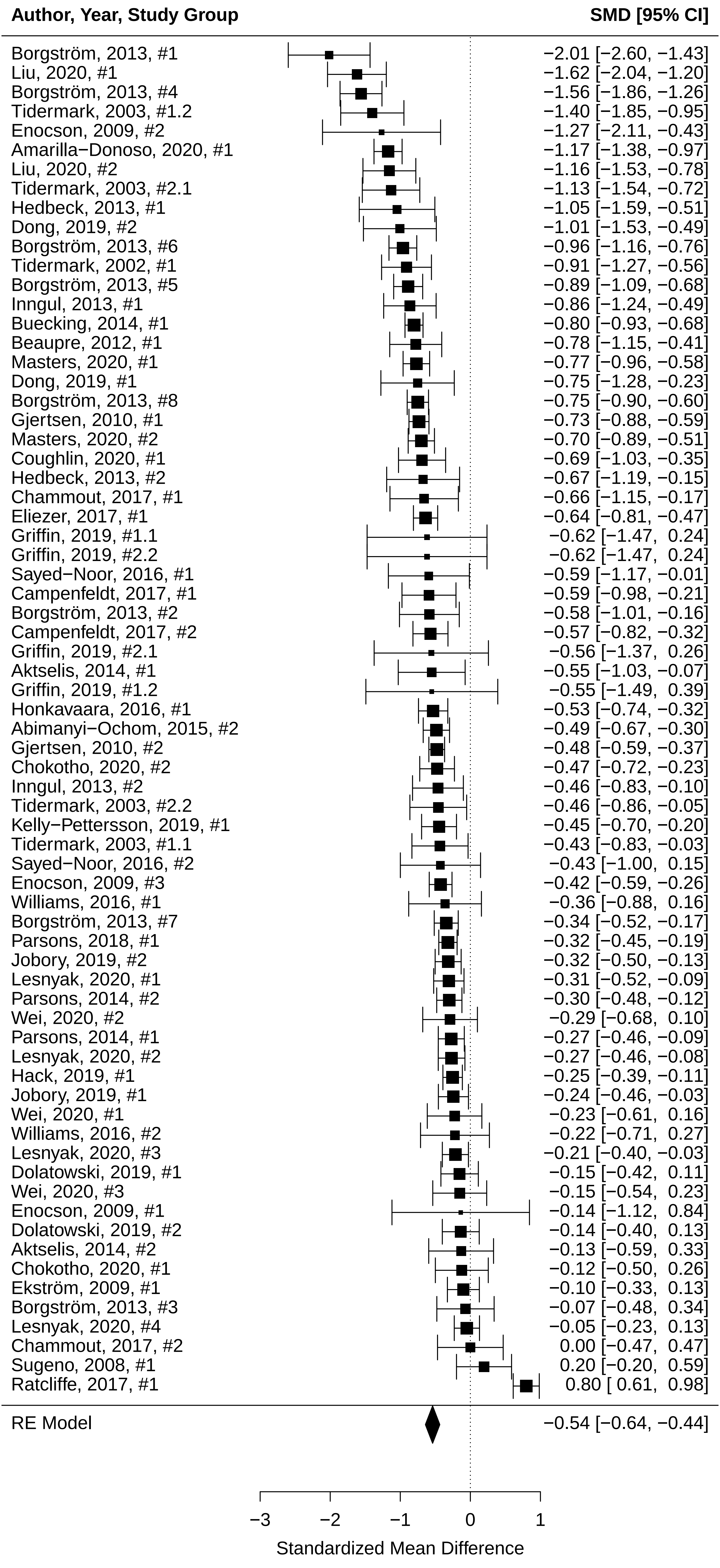
Author, Year, Study Group
SMD [95% CI]
Borgström, 2013, #1 −2.01 [−2.60, −1.43]
Liu, 2020, #1 −1.62 [−2.04, −1.20]
Borgström, 2013, #4 −1.56 [−1.86, −1.26]
Tidermark, 2003, #1.2 −1.40 [−1.85, −0.95]
Enocson, 2009, #2 −1.27 [−2.11, −0.43]
Amarilla−Donoso, 2020, #1 −1.17 [−1.38, −0.97]
Liu, 2020, #2 −1.16 [−1.53, −0.78]
Tidermark, 2003, #2.1 −1.13 [−1.54, −0.72]
Hedbeck, 2013, #1 −1.05 [−1.59, −0.51]
Dong, 2019, #2 −1.01 [−1.53, −0.49]
Borgström, 2013, #6 −0.96 [−1.16, −0.76]
Tidermark, 2002, #1 −0.91 [−1.27, −0.56]
Borgström, 2013, #5 −0.89 [−1.09, −0.68]
Inngul, 2013, #1 −0.86 [−1.24, −0.49]
Buecking, 2014, #1 −0.80 [−0.93, −0.68]
Beaupre, 2012, #1 −0.78 [−1.15, −0.41]
Masters, 2020, #1 −0.77 [−0.96, −0.58]
Dong, 2019, #1 −0.75 [−1.28, −0.23]
Borgström, 2013, #8 −0.75 [−0.90, −0.60]
Gjertsen, 2010, #1 −0.73 [−0.88, −0.59]
Masters, 2020, #2 −0.70 [−0.89, −0.51]
Coughlin, 2020, #1 −0.69 [−1.03, −0.35]
Hedbeck, 2013, #2 −0.67 [−1.19, −0.15]
Chammout, 2017, #1 −0.66 [−1.15, −0.17]
Eliezer, 2017, #1 −0.64 [−0.81, −0.47]
Griffin, 2019, #1.1 −0.62 [−1.47, 0.24]
Griffin, 2019, #2.2 −0.62 [−1.47, 0.24]
Sayed−Noor, 2016, #1 −0.59 [−1.17, −0.01]
Campenfeldt, 2017, #1 −0.59 [−0.98, −0.21]
Borgström, 2013, #2 −0.58 [−1.01, −0.16]
Campenfeldt, 2017, #2 −0.57 [−0.82, −0.32]
Griffin, 2019, #2.1 −0.56 [−1.37, 0.26]
Aktselis, 2014, #1 −0.55 [−1.03, −0.07]
Griffin, 2019, #1.2 −0.55 [−1.49, 0.39]
Honkavaara, 2016, #1 −0.53 [−0.74, −0.32]
Abimanyi−Ochom, 2015, #2 −0.49 [−0.67, −0.30]
Gjertsen, 2010, #2 −0.48 [−0.59, −0.37]
Chokotho, 2020, #2 −0.47 [−0.72, −0.23]
Inngul, 2013, #2 −0.46 [−0.83, −0.10]
Tidermark, 2003, #2.2 −0.46 [−0.86, −0.05]
Kelly−Pettersson, 2019, #1 −0.45 [−0.70, −0.20]
Tidermark, 2003, #1.1 −0.43 [−0.83, −0.03]
Sayed−Noor, 2016, #2 −0.43 [−1.00, 0.15]
Enocson, 2009, #3 −0.42 [−0.59, −0.26]
Williams, 2016, #1 −0.36 [−0.88, 0.16]
Borgström, 2013, #7 −0.34 [−0.52, −0.17]
Parsons, 2018, #1 −0.32 [−0.45, −0.19]
Jobory, 2019, #2 −0.32 [−0.50, −0.13]
Lesnyak, 2020, #1 −0.31 [−0.52, −0.09]
Parsons, 2014, #2 −0.30 [−0.48, −0.12]
Wei, 2020, #2 −0.29 [−0.68, 0.10]
Parsons, 2014, #1 −0.27 [−0.46, −0.09]
Lesnyak, 2020, #2 −0.27 [−0.46, −0.08]
Hack, 2019, #1 −0.25 [−0.39, −0.11]
Jobory, 2019, #1 −0.24 [−0.46, −0.03]
Wei, 2020, #1 −0.23 [−0.61, 0.16]
Williams, 2016, #2 −0.22 [−0.71, 0.27]
Lesnyak, 2020, #3 −0.21 [−0.40, −0.03]
Dolatowski, 2019, #1 −0.15 [−0.42, 0.11]
Wei, 2020, #3 −0.15 [−0.54, 0.23]
Enocson, 2009, #1 −0.14 [−1.12, 0.84]
Dolatowski, 2019, #2 −0.14 [−0.40, 0.13]
Aktselis, 2014, #2 −0.13 [−0.59, 0.33]
Chokotho, 2020, #1 −0.12 [−0.50, 0.26]
Ekström, 2009, #1 −0.10 [−0.33, 0.13]
Borgström, 2013, #3 −0.07 [−0.48, 0.34]
Lesnyak, 2020, #4 −0.05 [−0.23, 0.13]
Chammout, 2017, #2 0.00 [−0.47, 0.47]
Sugeno, 2008, #1 0.20 [−0.20, 0.59]
Ratcliffe, 2017, #1 0.80 [0.61, 0.98]
RE Model −0.54 [−0.64, −0.44]
−3
−2
−1
0
1
Standardized Mean Difference
