# Supplementary material for: Effects of medical interventions on health-related quality of life in chronic disease – systematic review and meta-analysis of the 19 most common diagnoses
Source: Front Public Health. 2024 Feb 6;12:1313685. doi: 10.3389/fpubh.2024.1313685 (PMC10878130; doi:10.3389/fpubh.2024.1313685)
Supplement: Supplementary file 10 [file Data_Sheet_1.ZIP › Frontiers_Supplementary_Figures/Riecke et al._Fig.S1S_S82.pdf]

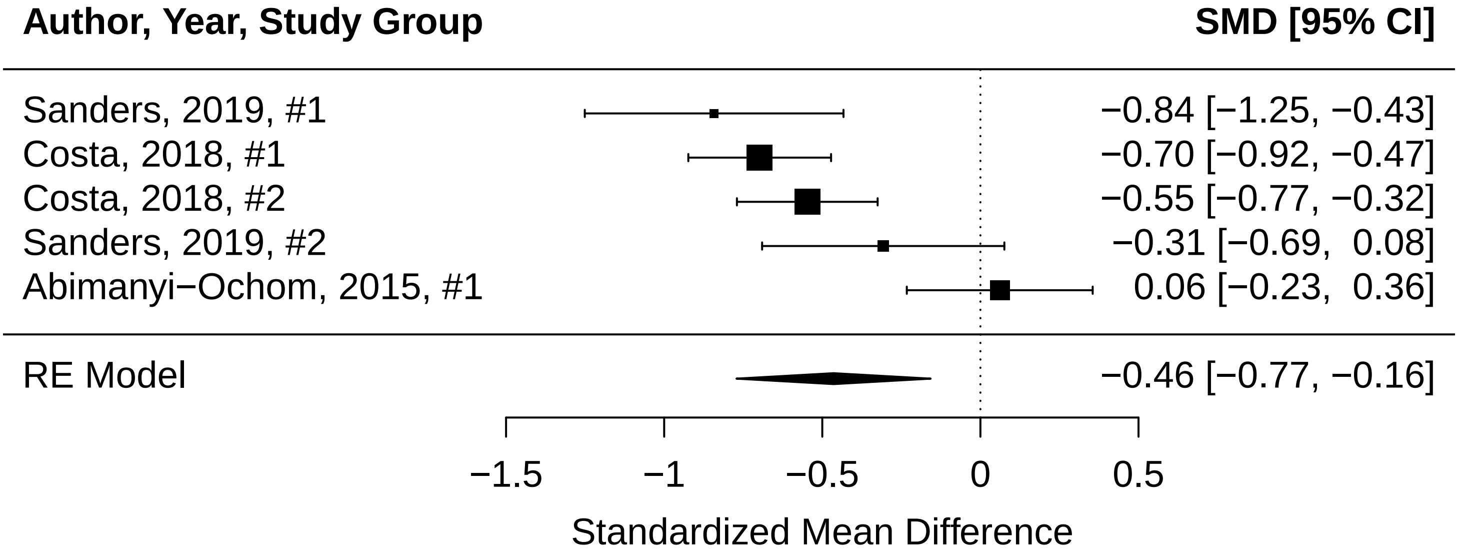

Author, Year, Study Group
SMD [95% CI]
Sanders, 2019, #1
−0.84 [−1.25, −0.43]
Costa, 2018, #1
−0.70 [−0.92, −0.47]
Costa, 2018, #2
−0.55 [−0.77, −0.32]
Sanders, 2019, #2
−0.31 [−0.69, 0.08]
Abimanyi−Ochom, 2015, #1
0.06 [−0.23, 0.36]
RE Model
−0.46 [−0.77, −0.16]
−1.5
−1
−0.5
0
0.5
Standardized Mean Difference
