# Supplementary figures and images for: Effects of medical interventions on health-related quality of life in chronic disease – systematic review and meta-analysis of the 19 most common diagnoses
Source: Front Public Health. 2024 Feb 6;12:1313685. doi: 10.3389/fpubh.2024.1313685 (PMC10878130; doi:10.3389/fpubh.2024.1313685)

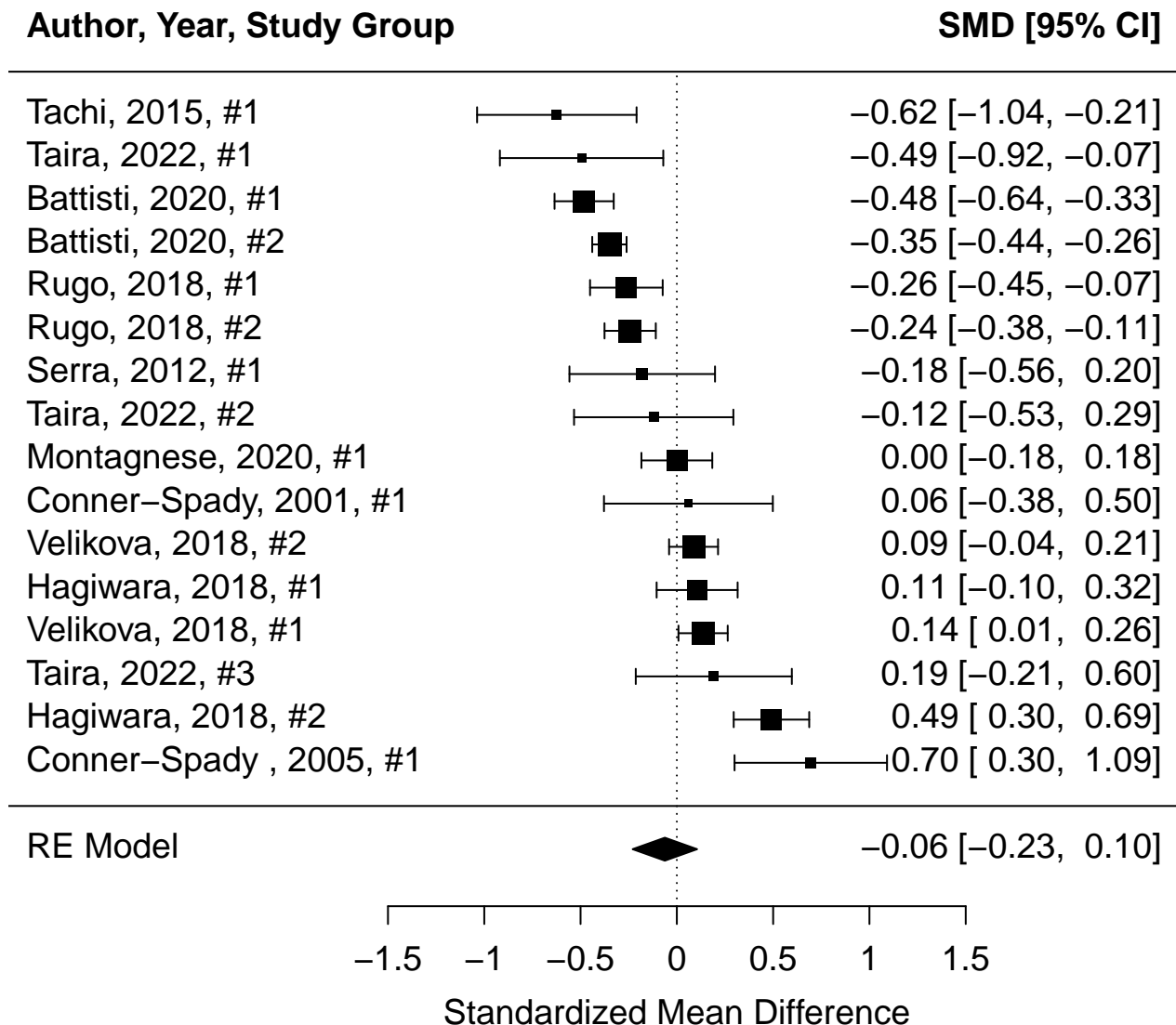

Supplement: Supplementary file 10 [file Data_Sheet_1.ZIP › Frontiers_Supplementary_Figures/Riecke et al._Fig.S1B_C50.pdf]

# Author, Year, Study Group

SMD [95% CI]

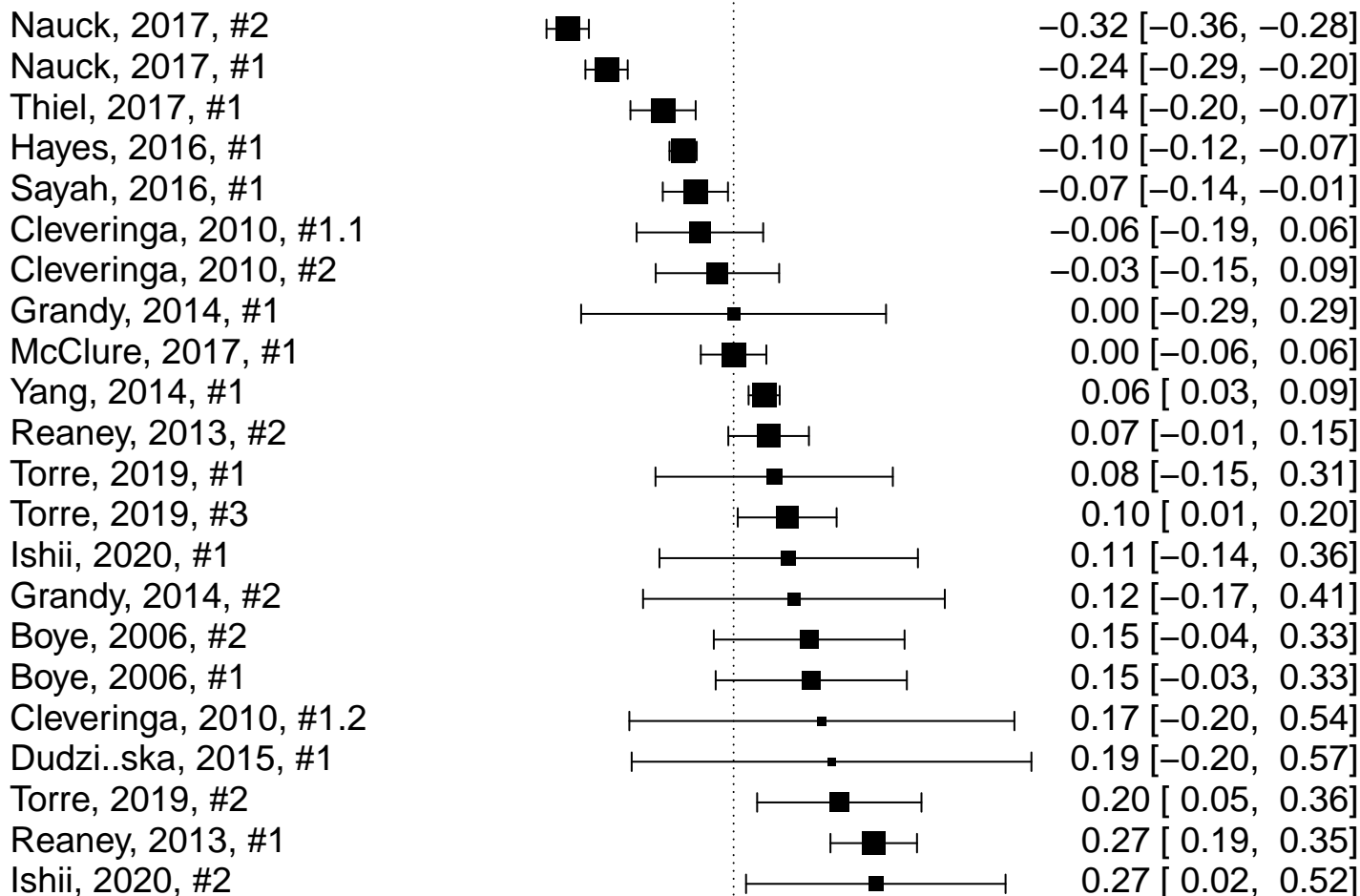

RE Model

0.02 [-0.05, 0.09]

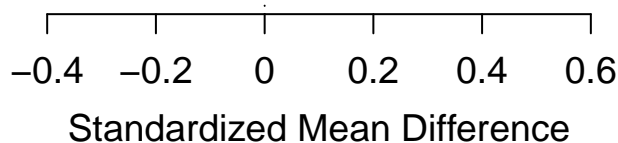

Supplement: Supplementary file 10 [file Data_Sheet_1.ZIP › Frontiers_Supplementary_Figures/Riecke et al._Fig.S1C_E11.pdf]

# Author, Year, Study Group

# SMD [95% CI]

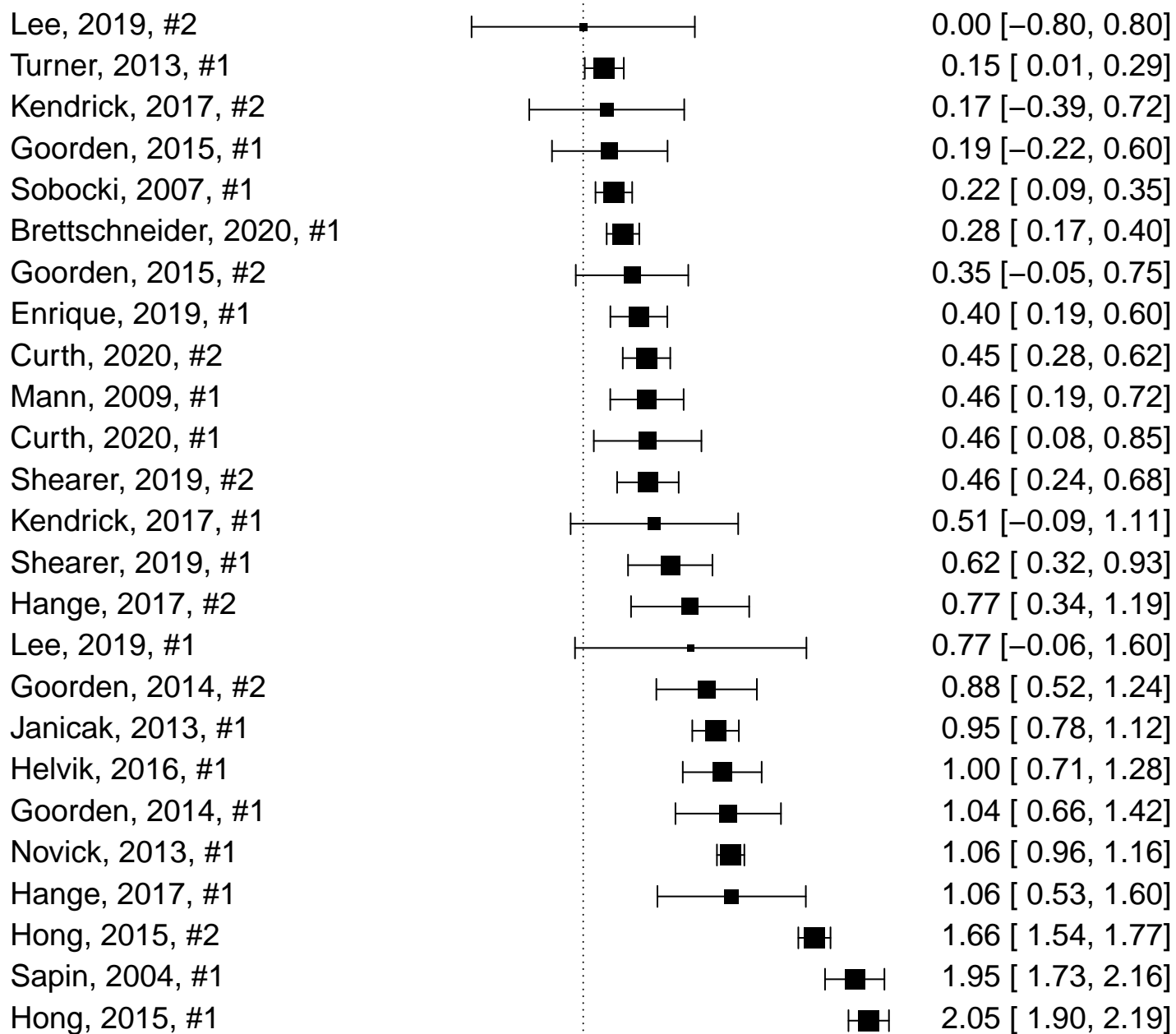

RE Model

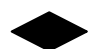

0.73 [ 0.51, 0.96]

-1 0 1 2 3

Standardized Mean Difference

Supplement: Supplementary file 10 [file Data_Sheet_1.ZIP › Frontiers_Supplementary_Figures/Riecke et al._Fig.S1D_F33.pdf]

**Author, Year, Study Group**

**SMD [95% CI]**

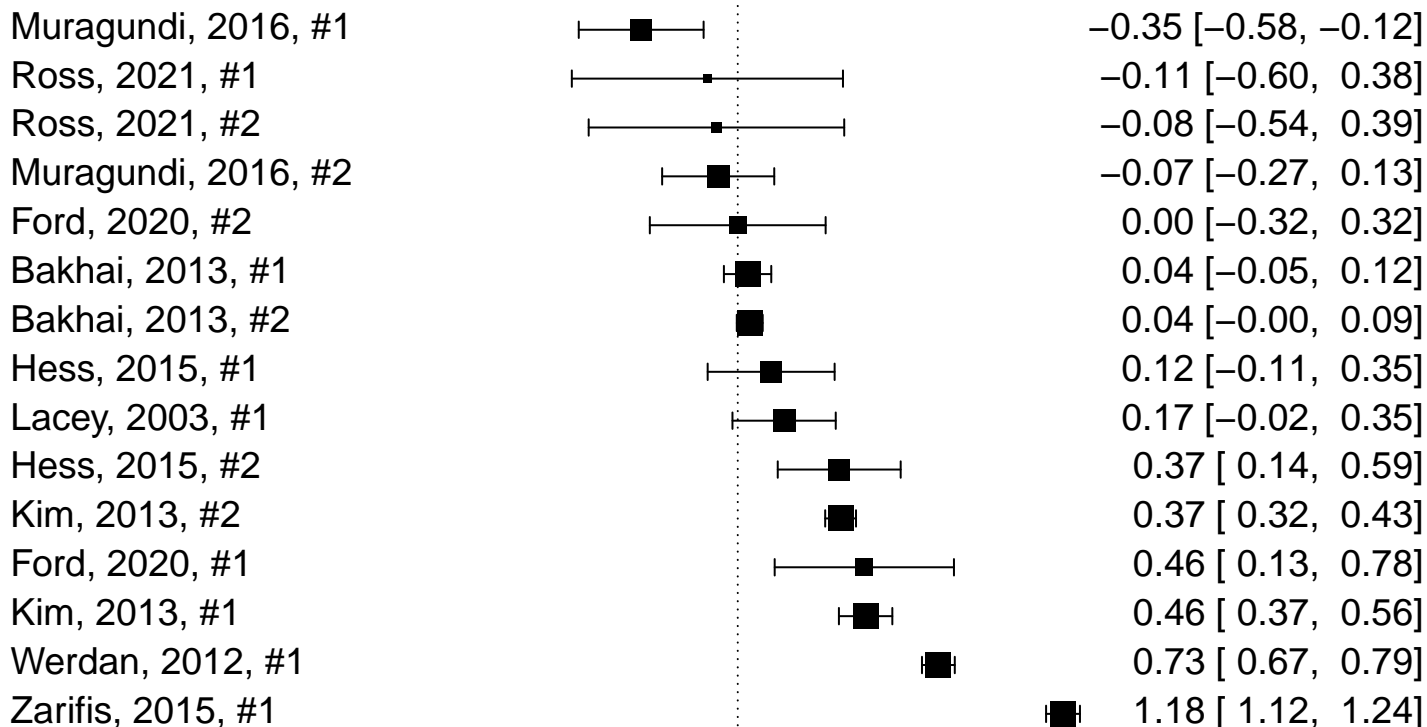

**RE Model**

**0.24 [0.04, 0.44]**

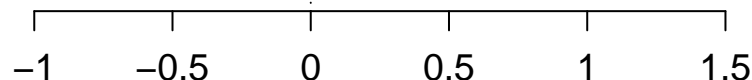

Standardized Mean Difference

Supplement: Supplementary file 10 [file Data_Sheet_1.ZIP › Frontiers_Supplementary_Figures/Riecke et al._Fig.S1F_I24.pdf]

**Author, Year, Study Group**

**SMD [95% CI]**

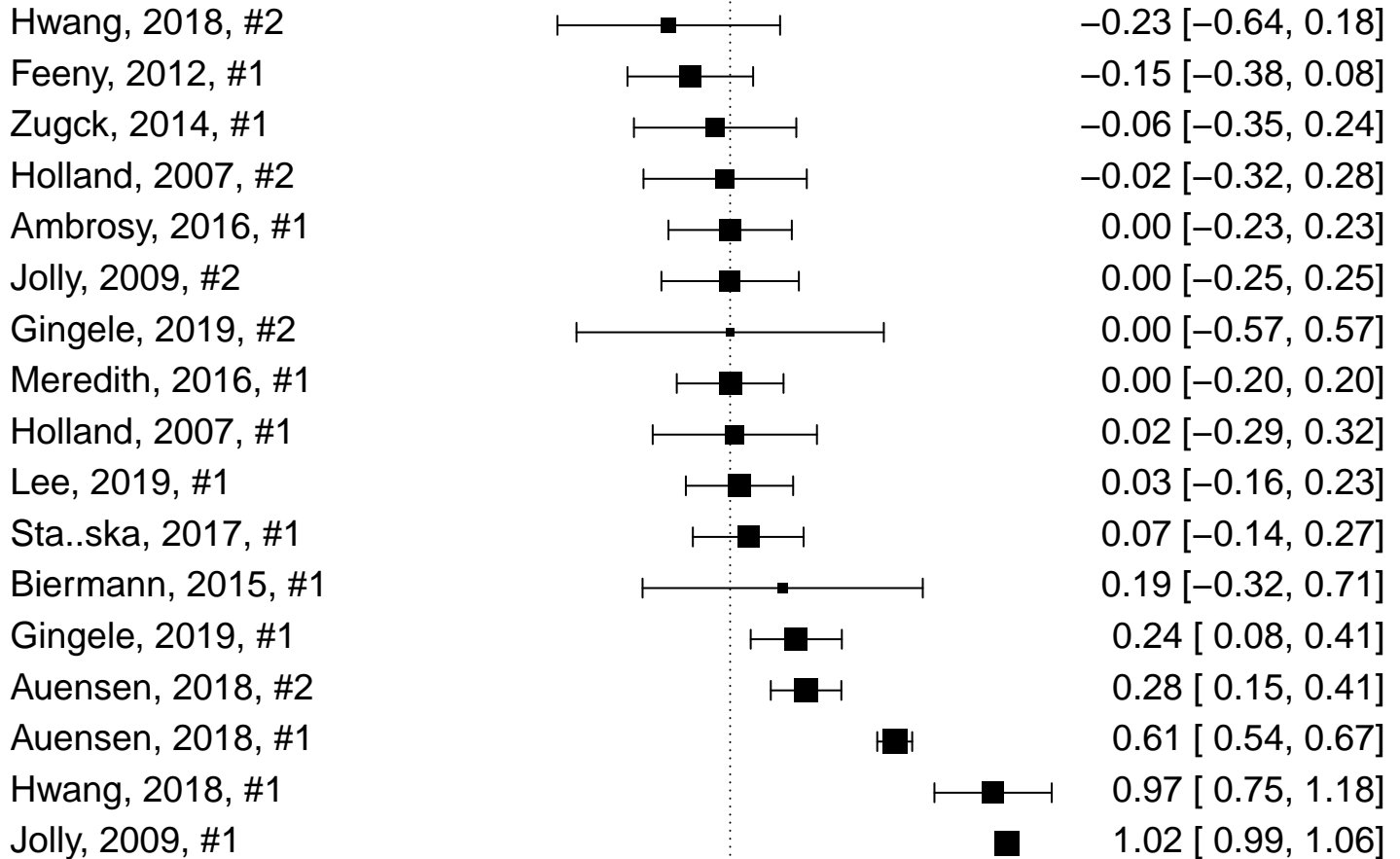

**RE Model**

**0.19 [ 0.01, 0.38]**

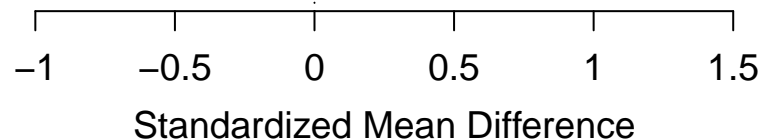

Supplement: Supplementary file 10 [file Data_Sheet_1.ZIP › Frontiers_Supplementary_Figures/Riecke et al._Fig.S1H_I50.pdf]

## Author, Year, Study Group

SMD [95% CI]

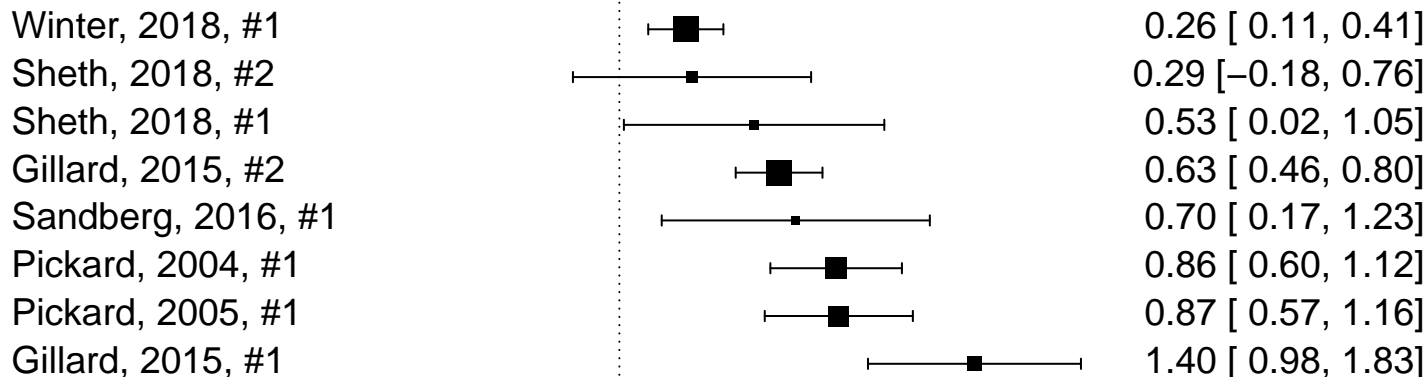

RE Model

0.69 [ 0.43, 0.94]

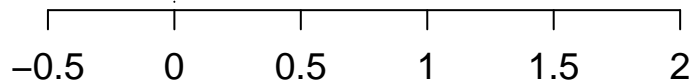

Standardized Mean Difference

Supplement: Supplementary file 10 [file Data_Sheet_1.ZIP › Frontiers_Supplementary_Figures/Riecke et al._Fig.S1I_I63.pdf]

**Author, Year, Study Group**

**SMD [95% CI]**

Ahn, 2020, #1

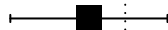

-0.08 [-0.25, 0.09]

Nguyen, 2019, #1

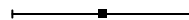

0.66 [ 0.47, 0.86]

RE Model

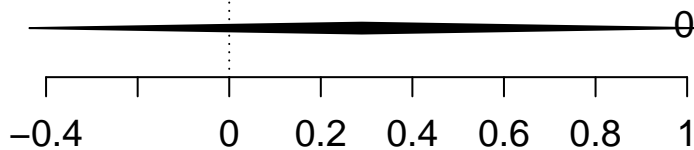

0.29 [-0.44, 1.02]

Standardized Mean Difference

Supplement: Supplementary file 10 [file Data_Sheet_1.ZIP › Frontiers_Supplementary_Figures/Riecke et al._Fig.S1J_J44.pdf]

**Author, Year, Study Group**

**SMD [95% CI]**

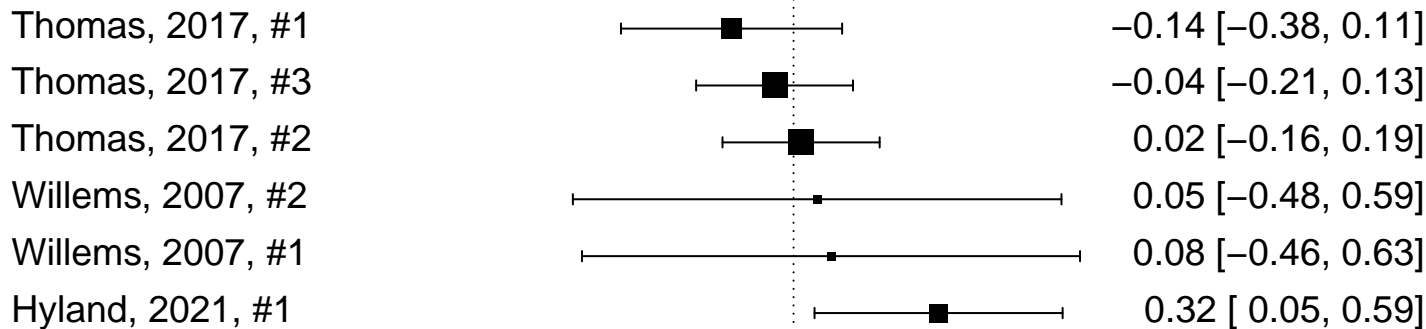

**RE Model**

**0.03 [-0.10, 0.15]**

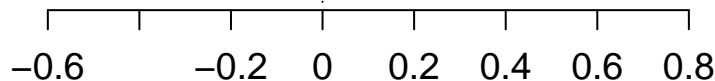

**Standardized Mean Difference**

Supplement: Supplementary file 10 [file Data_Sheet_1.ZIP › Frontiers_Supplementary_Figures/Riecke et al._Fig.S1K_J45.pdf]

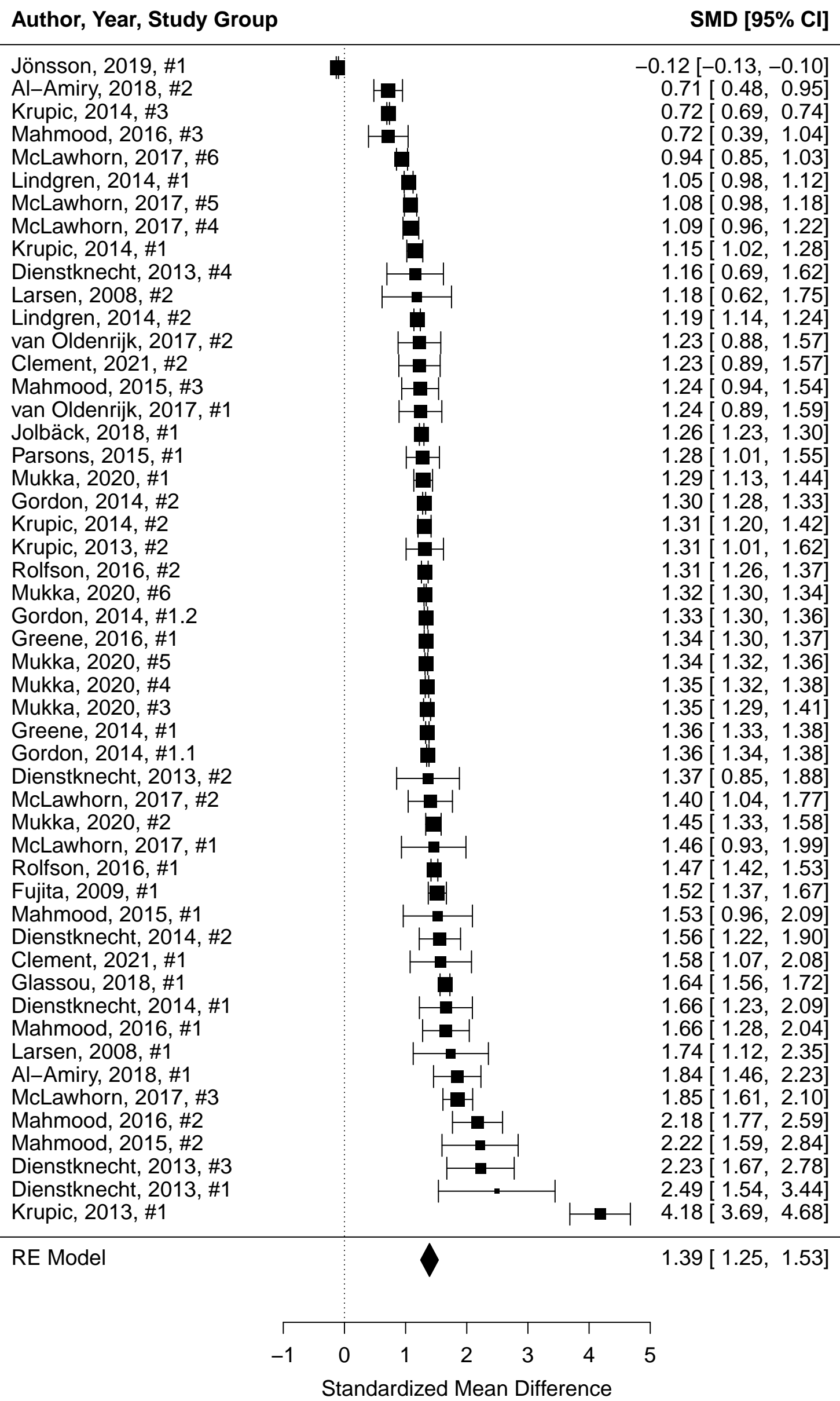

Supplement: Supplementary file 10 [file Data_Sheet_1.ZIP › Frontiers_Supplementary_Figures/Riecke et al._Fig.S1L_M16.pdf]

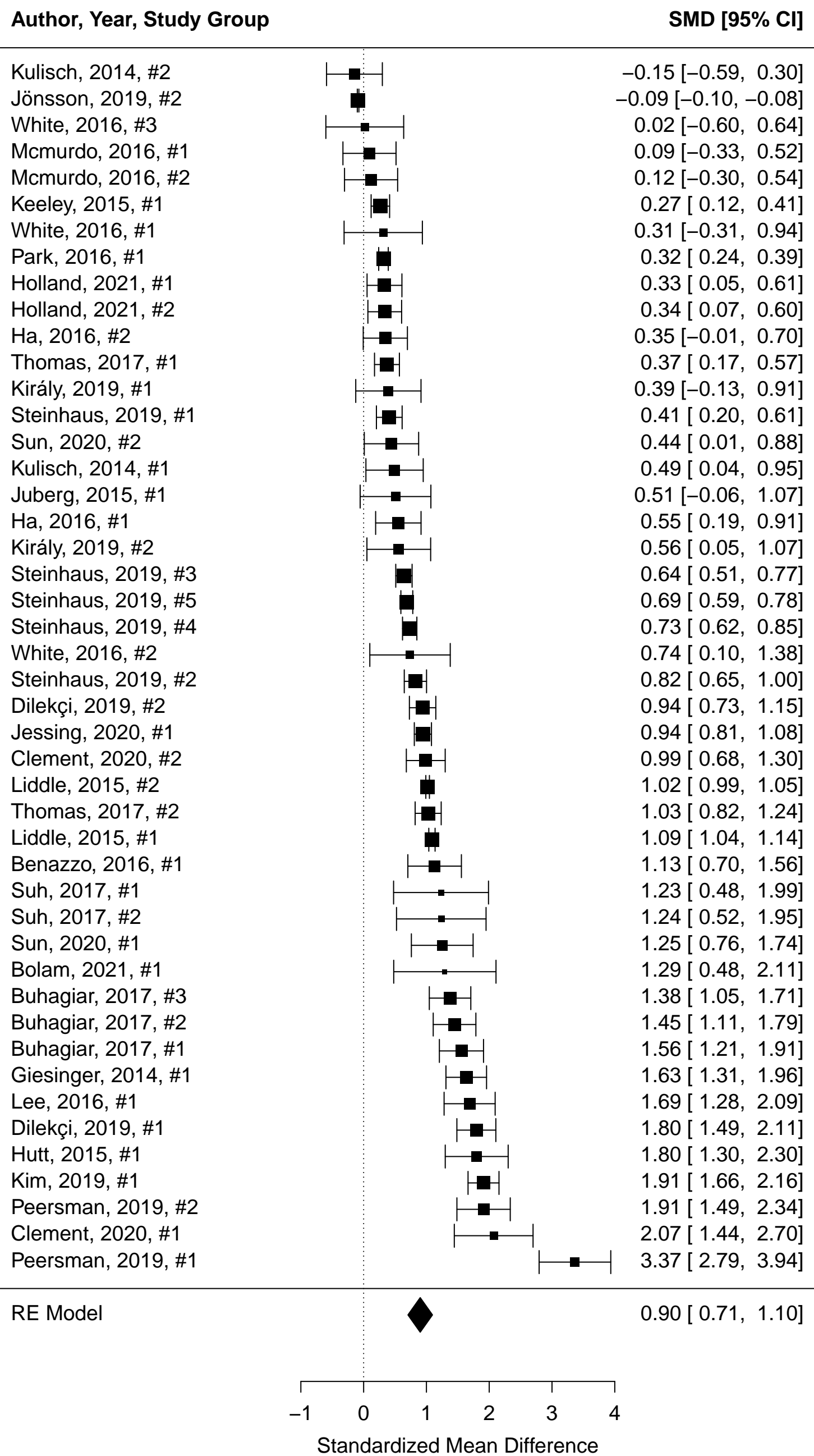

Supplement: Supplementary file 10 [file Data_Sheet_1.ZIP › Frontiers_Supplementary_Figures/Riecke et al._Fig.S1M_M17.pdf]

**Author, Year, Study Group**

**SMD [95% CI]**

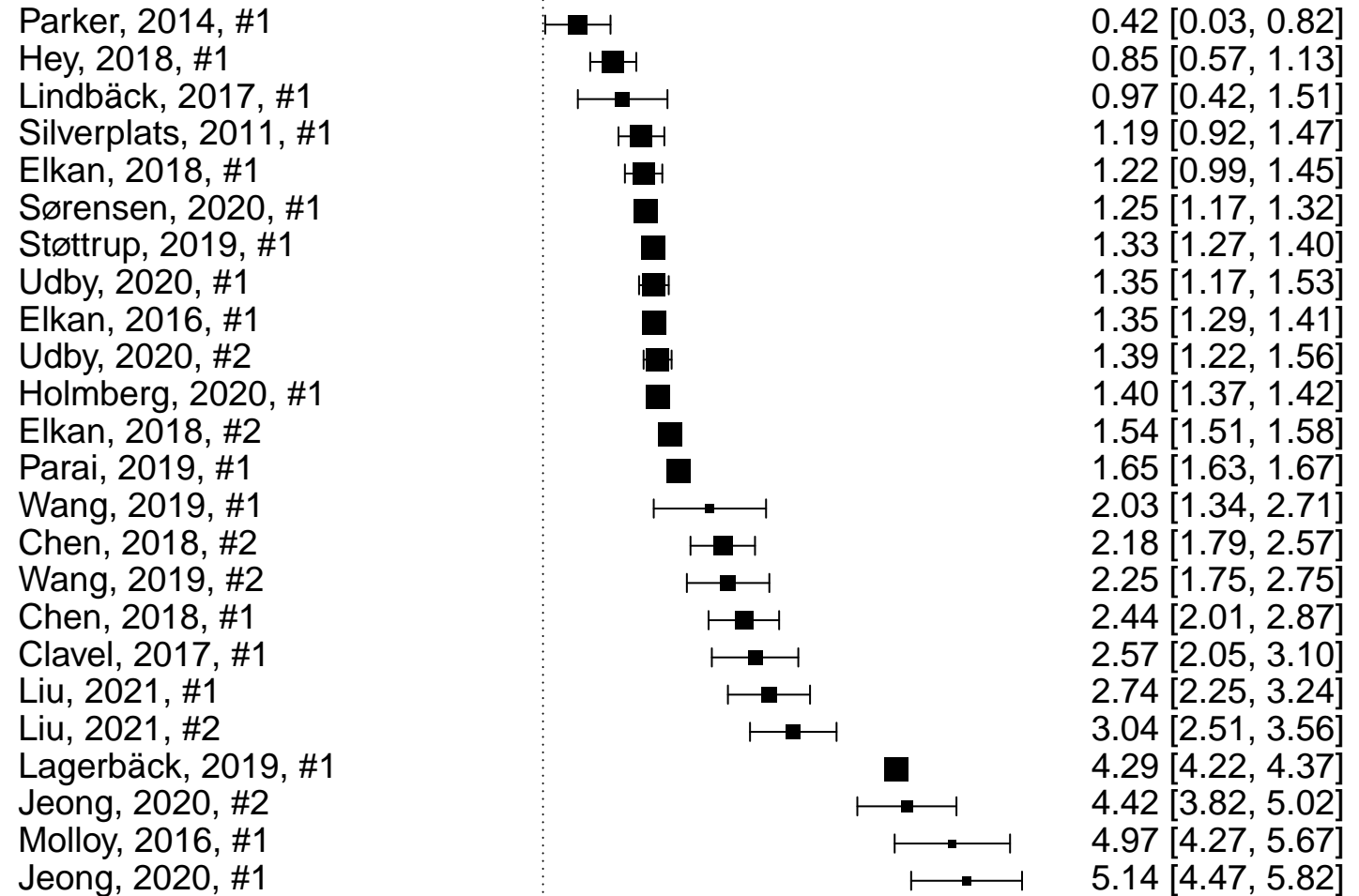

**RE Model**

**2.15 [1.63, 2.67]**

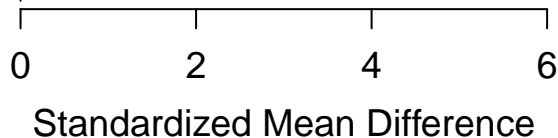

Supplement: Supplementary file 10 [file Data_Sheet_1.ZIP › Frontiers_Supplementary_Figures/Riecke et al._Fig.S1N_M51.pdf]

**Author, Year, Study Group**

**SMD [95% CI]**

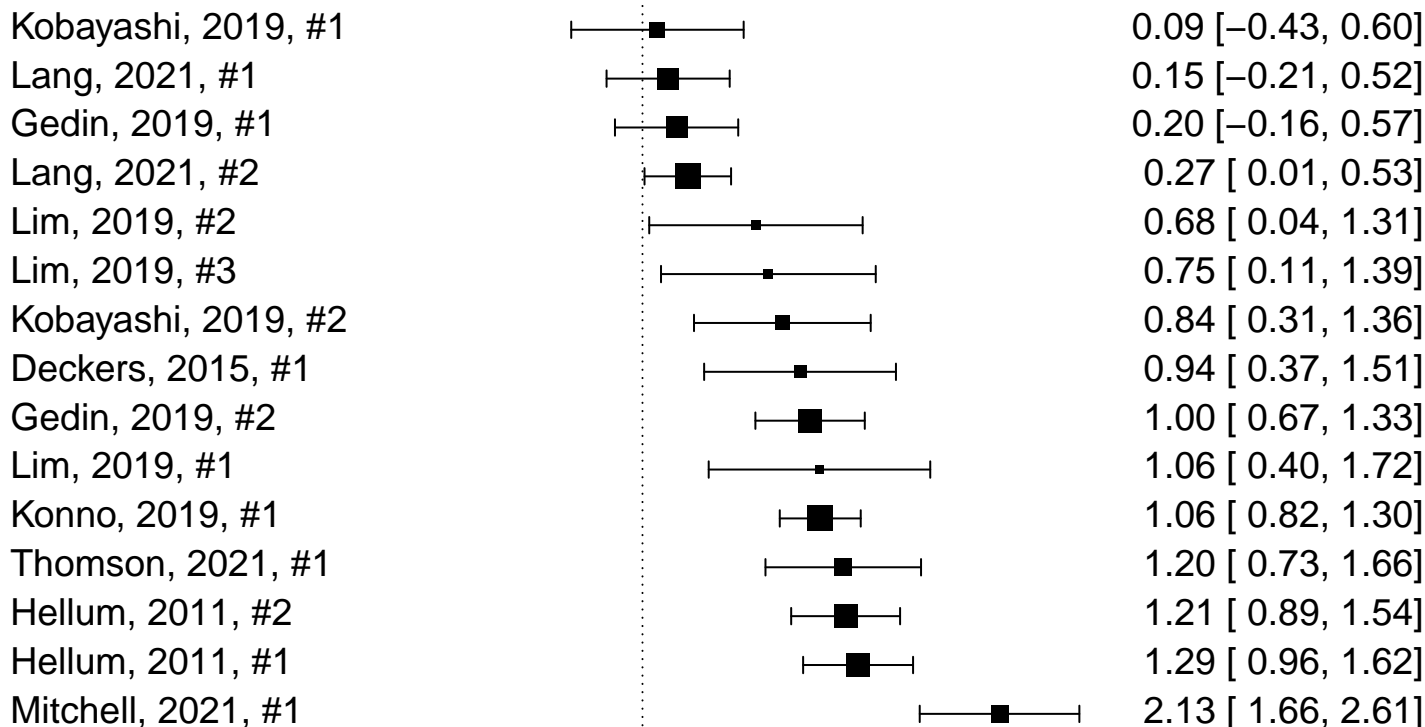

**RE Model**

**0.86 [ 0.58, 1.13]**

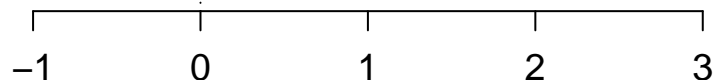

Standardized Mean Difference

Supplement: Supplementary file 10 [file Data_Sheet_1.ZIP › Frontiers_Supplementary_Figures/Riecke et al._Fig.S1O_M54.pdf]

PMID, Year

SMD [95% CI]

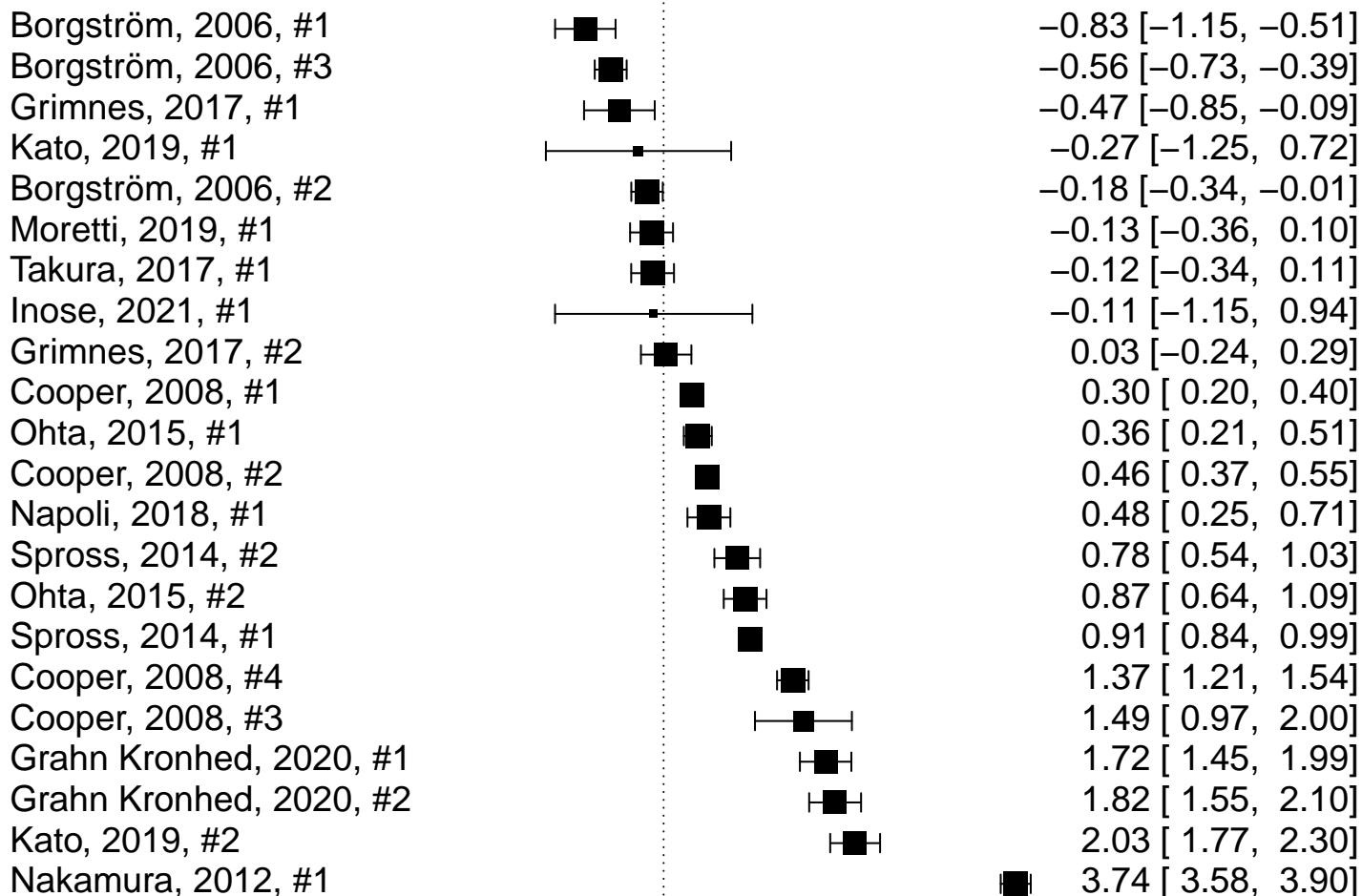

RE Model

0.64 [0.18, 1.09]

-2 -1 0 1 2 3 4

Standardized Mean Difference

Supplement: Supplementary file 10 [file Data_Sheet_1.ZIP › Frontiers_Supplementary_Figures/Riecke et al._Fig.S1P_M80,M81.pdf]

**Author, Year, Study Group**

**SMD [95% CI]**

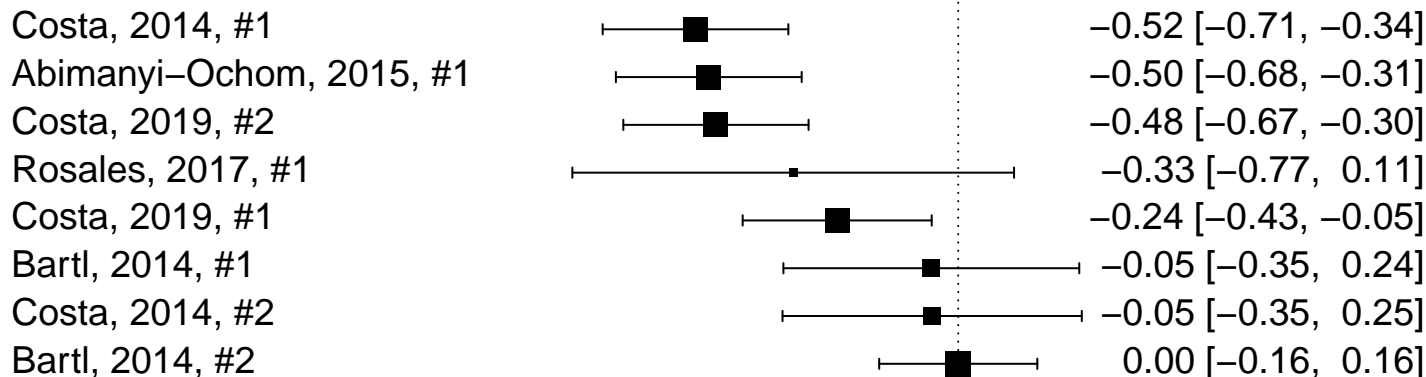

**RE Model**

**-0.28 [-0.44, -0.12]**

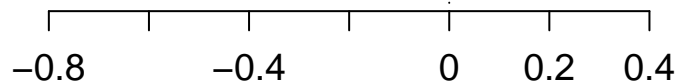

Standardized Mean Difference

Supplement: Supplementary file 10 [file Data_Sheet_1.ZIP › Frontiers_Supplementary_Figures/Riecke et al._Fig.S1Q_S52.pdf]
